# Redox States of Protein Cysteines in Pathways of Protein Turnover and Cytoskeleton Dynamics Are Changed with Aging and Reversed by Slc7a11 Restoration in Mouse Lung Fibroblasts

**DOI:** 10.1155/2020/2468986

**Published:** 2020-06-07

**Authors:** Yuxuan Zheng, Michael L. Merchant, Tom J. Burke, Jeffrey D. Ritzenthaler, Ming Li, Adam E. Gaweda, Frederick W. Benz, Jesse Roman, Walter H. Watson

**Affiliations:** ^1^Department of Pharmacology and Toxicology, University of Louisville School of Medicine, Louisville, KY 40202, USA; ^2^Department of Medicine, Division of Nephrology and Hypertension, University of Louisville School of Medicine, Louisville, KY 40202, USA; ^3^Department of Medicine, Division of Gastroenterology, Hepatology and Nutrition, University of Louisville School of Medicine, Louisville, 40202 KY, USA; ^4^Department of Medicine, Division of Pulmonary, Allergy and Critical Care Medicine, Sidney Kimmel Medical College and Jane and Leonard Korman Respiratory Institute, Thomas Jefferson University, Philadelphia, 19107 PA, USA

## Abstract

Slc7a11 is the key component of system X_c_^−^, an antiporter that imports cystine (CySS) and exports glutamate. It plays an important role in cellular defense against oxidative stress because cysteine (Cys), reduced from CySS, is used for and limits the synthesis of glutathione (GSH). We have shown that downregulation of Slc7a11 is responsible for oxidation of extracellular Cys/CySS redox potential in lung fibroblasts from old mice. However, how age-related change of Slc7a11 expression affects the intracellular redox environment of mouse lung fibroblasts remains unexplored. The purpose of this study is to evaluate the effects of aging on the redox states of intracellular proteins and to examine whether Slc7a11 contributes to the age-dependent effects. Iodoacetyl Tandem Mass Tags were used to differentially label reduced and oxidized forms of Cys residues in primary lung fibroblasts from young and old mice, as well as old fibroblasts transfected with Slc7a11. The ratio of oxidized/reduced forms (i.e., redox state) of a Cys residue was determined via multiplexed tandem mass spectrometry. Redox states of 151 proteins were different in old fibroblasts compared to young fibroblasts. Slc7a11 overexpression restored redox states of 104 (69%) of these proteins. Ingenuity Pathway Analysis (IPA) showed that age-dependent Slc7a11-responsive proteins were involved in pathways of protein translation initiation, ubiquitin-proteasome-mediated degradation, and integrin-cytoskeleton-associated signaling. Gene ontology analysis showed cell adhesion, protein translation, and organization of actin cytoskeleton were among the top enriched terms for biological process. Protein-protein interaction network demonstrated the interactions between components of the three enriched pathways predicted by IPA. Follow-up experiments confirmed that proteasome activity was lower in old cells than in young cells and that upregulation of Slc7a11 expression by sulforaphane restored this activity. This study finds that aging results in changes of redox states of proteins involved in protein turnover and cytoskeleton dynamics, and that upregulating Slc7a11 can partially restore the redox states of these proteins.

## 1. Introduction

Aging has been proposed as a consequence of the failure of redox networks to sustain biological functions [1]. This redox theory of aging accounts for several hallmarks of aging, including altered intercellular communication, loss of proteostasis, epigenetic alterations, and mitochondrial dysfunction [1, 2], because each of these is sensitive to changes in the redox state of one or more of its constituent components. One convenient way to assess changes in systemic redox states is to measure the redox potential (E_h_) of the cysteine/cystine (Cys/CySS) thiol/disulfide redox couple (E_h_(Cys/CySS)). Human plasma typically has an E_h_(Cys/CySS) of about -80 mV, and cells grown in culture condition their media to this same value [3, 4]. In fact, most cell types that have been studied return their media to -80 mV within 4 hours of media change [4–6]. As we age, plasma E_h_(Cys/CySS) becomes progressively more oxidized [7], and cultured old cells condition their media to more oxidized values when compared to young cells [4]. Thus, aging is associated with a disrupted redox environment.

We have found that the expression of Slc7a11, the light chain of the CySS transporter system X_c_^−^, is decreased in old mouse lung fibroblasts compared to that of young fibroblasts [4, 8]. Pharmacological induction or genetic overexpression of Slc7a11 restored extracellular E_h_(Cys/CySS), confirming that Slc7a11 is responsible for controlling extracellular E_h_(Cys/CySS) in primary mouse lung fibroblasts [8]. Previous studies showed extracellular E_h_(Cys/CySS) was associated with a variety of biological processes and diseases. For example, medium with oxidized E_h_(Cys/CySS) induced the expression of TGF-*β* and fibronectin and stimulated the proliferation of mouse lung fibroblasts [9]. Oxidized extracellular E_h_(Cys/CySS) also promoted mitochondrial thioredoxin-2 oxidation and induced reactive oxygen species (ROS) production in aortic endothelial cells [10]. In C6 glial cells, oxidized E_h_(Cys/CySS) activated metab

otropic glutamate receptor 5 and its downstream signaling pathways [11]. Plasma E_h_(Cys/CySS) was more oxidized in patients with chronic obstructive pulmonary disease compared to that in patients with normal lung function [12]. Thus, Slc7a11 has the potential to influence important intracellular signaling pathways and biological functions via controlling the extracellular redox environment.

Once transported into cells by system X_c_^−^, CySS is reduced to Cys, which is the rate-limiting component for GSH synthesis. GSH and Cys have the capacity to influence the redox states of individual intracellular proteins. Proteins contain cysteines that can be reversibly oxidized to disulfides or sulfenic acids. If a redox-sensitive cysteine is located in a catalytic or allosteric regulatory site, or a protein-protein or protein-DNA interaction domain, its redox state can determine the function of that protein. Many protein kinases have been shown to contain redox-sensitive cysteines. For example, oxidation of Cys797 to a sulfenic acid in the active site of EGFR increased its tyrosine kinase activity [13]. In contrast, intramolecular disulfide bond between Cys297 and Cys311 and oxidation of Cys124 to a sulfenic acid inhibited the kinase activity of Akt2, demonstrating that predicting specific changes of biological function resulting from alterations in the redox states of cysteines is difficult, thereby requiring evaluation on a case-by-case basis and requiring experimental confirmation [14, 15]. Furthermore, redox changes can affect structural proteins that contain redox-reactive thiols. In primary mouse lung fibroblasts, for example, cadmium exposure induced the oxidation of cysteines of actin and actin-associated cytoskeleton proteins, thus changing actin dynamics via increasing filamentous actin formation and transporting destrin from cytoplasm to nucleus [16].

The large number and wide distribution of protein cysteines increase the functional flexibility of the proteome and play important roles in signal transduction, aging, and disease [17]. Redox proteomics can be used to define the redox states of all protein cysteines under a given set of conditions. Various redox proteomics methods have been developed, but they share some common features. In general, these methods rely on differentially labeling reduced and oxidized cysteines and separating the 2 forms from each other, followed by the quantification of each form [18]. Ideally, the same chemical species should be used to label each form to minimize differences arising from chemical reactivity. Isotopic differences can be used to distinguish the labels by mass spectrometry. Iodo-TMT tags are well-suited to this purpose because they are available in several different masses, allowing multiplexing of samples. This approach was recently used to study the change of redox states of proteins in brown adipose tissue of mice exposed to cold temperature [19]. We have adapted this method for labeling reduced and oxidized cysteines to compare redox states between murine young and old lung fibroblasts, and old fibroblasts with Slc7a11 overexpression.

Currently, there is no information about how Slc7a11 affects the redox state and function of the cysteine proteome. Furthermore, how aging changes the redox state of the cysteine proteome in primary mouse lung fibroblasts is unknown. This study aims at identifying intracellular protein cysteines whose redox states are age-dependent and at exploring the potential biological functions of these cysteine-containing proteins. Considering that low Slc7a11 expression in old fibroblasts is responsible for the change of the extracellular redox environment with aging, we also aim at exploring whether and to what extent restoration of Slc7a11 expression would reverse the age-dependent changes of intracellular redox environment and signaling pathways.

## 2. Materials and Methods

### 2.1. Reagents

All reagents were purchased from Sigma-Aldrich (St. Louis, MO) or Corning (Manassas, VA) unless otherwise specified.

### 2.2. Culture of Primary Mouse Lung Fibroblasts

Primary lung fibroblasts were isolated from young (3 months) or old (24 months) female C57BL/6 mice as described previously [20, 21]. Animal maintenance and procedures of animal experiments were approved by the Institutional Animal Care and Use Committee of the University of Louisville. After isolation, fibroblasts were cultured in DMEM supplemented with 10% fetal bovine serum (FBS) and 1% antibiotic-antimycotic solution in a humidified incubator with 5% CO_2_. Fibroblasts between passage numbers 10 and 15 were used in the experiments.

### 2.3. Preparation of Redox Media with E_h_(Cys/CySS) of 0 mV

0 mV redox media were prepared by adding 99.75 *μ*M CySS and 0.5 *μ*M Cys to cysteine-free and FBS-free DMEM. All redox media were freshly prepared and used for fibroblast incubation immediately. Note that a typical preparation of DMEM contains 200 *μ*M CySS and, therefore, has a more positive (more oxidizing) redox potential than the 0 mV media used here.

### 2.4. Overexpression of Slc7a11 Expression

Plasmid encoding mouse Slc7a11 was purchased from Origene Technologies Inc. (Rockville, MD). Two micrograms of plasmid were used to transfect 1 million primary lung fibroblasts from an old mouse via electroporation. The program of U-023 on the Nucleofector™ 2b Device (Lonza, Allendale, NJ) and the protocol of the Amaxa™ Basic Nucleofector™ Kit for Primary Mammalian Fibroblasts (Lonza, Allendale, NJ) were applied for electroporation. Our previous studies showed that this electroporation protocol had no effect on the extracellular E_h_(Cys/CySS) maintained by either young or old fibroblasts [8].

### 2.5. Experimental Design

Using 6-well plates, 300,000 cells of the following types were plated in triplicate: primary lung fibroblasts from a young female mouse, primary lung fibroblasts from an old mouse, and old mouse fibroblasts transfected with Slc7a11 as described in Section 2.4. After transfection, cells were allowed to recover in antibiotic-free DMEM with 10% FBS. After 24 hours, the media in all wells were changed to 0 mV redox media and incubated for 4 hours. This final media change to 0 mV media was included to (1) reproduce the conditions of our previous studies [8] and (2) verify that the young and old cells had different homeostatic extracellular redox potentials and document the effect of Slc7a11 overexpression. Finally, media and cells were collected for measuring extracellular E_h_(Cys/CySS) via HPLC and redox states of cysteine residues of intracellular proteins, respectively.

### 2.6. Media Derivatization and High-Performance Liquid Chromatography (HPLC) Analysis

Detailed procedures were described previously [8]. Briefly, the media were transferred to a tube containing the same amount of ice-cold solution consisting of 10% (*w*/*v*) perchloric acid, 0.2 M boric acid, and 20 *μ*M *γ*-glutamyl glutamate [22]. Then, the media were derivatized with iodoacetic acid and dansyl chloride for HPLC analysis (Waters Corporation, Milford, MA) [23]. The calculation of E_h_(Cys/CySS) was based on the Nernst equation for pH 7.4: E_h_(Cys/CySS) = −250 + 30∗log([CySS]/[Cys]^2^) [3]. Although this equation was derived to describe systems at equilibrium, its application to biological (nonequilibrium) conditions provides a useful indication of changes in redox potentials and allows comparisons of different redox couples and redox environments [3, 24].

### 2.7. Labeling of Reduced and Oxidized Cysteine Residues of Intracellular Proteins with Different Iodoacetyl Tandem Mass Tags (iodoTMTs)

Our labeling method was developed based on the isobaric iodoTMT 6plex reagent that was recently used to study the redox proteome of E. coli [25]. Each iodoTMT label contains a thiolate specific-reactive group for labeling reduced thiols, a mass reporter with different numbers of isotope positions for distinguishing different tags in the mass spectrometry, and a mass normalizer for balancing the molecular weight. Proteins of fibroblasts in each well were collected and frozen in 300 *μ*l of 10% trichloroacetic acid (TCA) before labeling. After thawing, proteins were centrifuged at 16,000 g at 4°C for 15 min. The protein pellet was washed with ice-cold acetone. Then, the protein pellet was dissolved in 200 *μ*l of denaturing buffer consisting of 200 mM Tris, 1 mM EDTA, and 0.1% (*w*/*v*) SDS, pH 8.5. Immediately thereafter, 100 *μ*l of dissolved proteins was added to a tube containing one of six iodoTMT reagents freshly dissolved in 10 *μ*l methanol and incubated for 1 hour at 37°C. This step labels the reduced cysteines. Proteins were precipitated once again in TCA and washed with acetone to remove unincorporated iodoTMT reagent. Proteins were dissolved in 100 *μ*l of denaturing buffer, and 1 *μ*l of 500 mM TCEP was added to reduce reversibly oxidized protein cysteine residues. The newly-reduced cysteines were then labeled by adding the second iodoTMT label and incubating for 1 hour at 37°C. Again, proteins were precipitated with TCA and washed with acetone to remove unincorporated iodoTMT. Finally, double-labeled protein pellet was dissolved in 60 *μ*l of denaturing buffer. Total protein concentration was measured by Bio-Rad DC protein assay using gamma globulin as the standard (Bio-Rad, Hercules, CA).

### 2.8. Determination of Relative Protein Abundance by 10plex-TMT Labeling and Liquid Chromatography-Tandem Mass Spectrometry (LC-MS/MS)

In a separate experiment, two young and two old fibroblast samples were analyzed for relative protein quantification. Protein samples, 50 *μ*g each, were loaded to filters with molecular weight cut-off at 10 kDa to exchange buffer to 0.1 M ammonium bicarbonate through ultrafiltration. Then, the samples were reduced with dithiothreitol, alkylated with iodoacetamide, and digested with trypsin at 37°C overnight. The digested samples were desalted with the C_18_ column, dried by speedvac, and labeled with TMT 10plex tags (127 N and 128 N for young fibroblasts samples and 129 N and 130 N for old fibroblasts samples). To ensure the completion of labeling, the samples were labeled twice with the reagents at 50°C for 1 hour. After labeling, the reaction was quenched with hydroxylamine, and the samples were mixed and desalted with a C_18_ column, dried by speedvac, and fractionated by high-pH LC. The high-pH fractionation was performed on a Beckman System Gold HPLC (Beckman Coulter, Brea, CA) with an XBridge C_18_ 5 *μ*m 3.0 × 150 mm column (Waters, Milford, MA) by a 75 min binary solvent gradient (10 mM ammonium formate/5% ACN at pH 10 and 10 mM ammonium formate/90% ACN at pH 10). During LC separation, elute from 5 to 66.2 min was collected as fifty-one 1.2 min fractions and these fractions were concatenated into 17 fractions (fraction #2 to #18). Also, elute from 0 to 5 min and elute from 66.2 to 75 min were collected as fraction #1 and #19, respectively. These fractions were dried by speedvac, dissolved in 75% ACN/0.1% FA, filtered through 0.2 *μ*m filters, dried by speedvac, and reconstituted in 1% formic acid before LC-MS analysis. The fractionated samples were analyzed by an Easy-nLC 1000 and Orbitrap Elite MS system (Thermo Scientific). Peptides in samples were trapped on a *μ*-precolumn and then transferred to and separated on an in-house packed C_18_ analytic column (particle size 3.6 *μ*m, 100 *μ*m i.d.×125 mm) with a 120 min binary solvent gradient (0.1% formic acid and acetonitrile with 0.1% formic acid). Eluate from the column was directly ionized by a nanospray source and analyzed by the mass spectrometer in DDA mode. The mass resolution was set to 60,000 and 30,000 for MS and MS/MS, respectively. AGC, isolation window, and off set were set to 2e5, 2 *m*/*z*, and 0.5 *m*/*z*. Acquired data were processed by Proteome Discoverer (PD v1.4.1.14) with Sequest HT and Mascot (v.2.5.1) search engines and mouse protein database from NCBI (downloaded on 8/30/2016). Data were searched considering two missed tryptic cleavages, static modifications of amino groups at N-terminal of all peptides and lysine residues by TMT 10plex tag, and cysteine residues by carbamidomethylation, as well as dynamic modification of methionine oxidation were applied in the search. Match tolerances were set to 20 ppm, 5 mDa, and 20 ppm for precursor, fragment, and report ions, respectively. The target-decoy PSM validator node in PD v1.4 was used to estimate the false discovery rates (FDR) for peptide identifications. High confidence peptide sequence assignments were used at the ≤1% FDR and based on Sequest HT Xcorr scores greater than 2.1 for +2 and +3 charge states; Mascot significance threshold probability equal to or lower than 0.0045. A minimum of 2 high confidence (1% FDR-) peptide matches were required for protein identification, and proteins matched with the same peptides were reported as a protein group. For relative quantification, the intensity of report ions from peptides specific to the protein group was used to calculate the relative abundance of identified proteins. Because each group contained only 2 replicates, the statistical significance of relative abundance differences between old and young cells was not assessed. Rather, a fold-change cut-off of greater than 2 was applied.

### 2.9. Digestion, Affinity Purification, and LC-MS/MS Analysis of iodoTMT Labeled Tryptic Peptides

**Figure 1:**
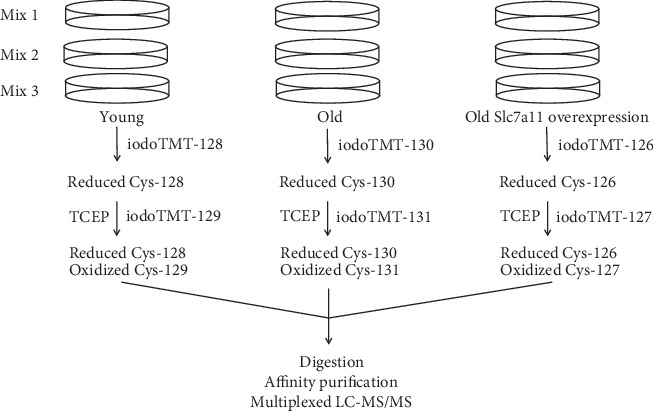
Schematic flow chart of labeling redox-sensitive cysteines in peptides from young, old, and old Slc7a11-overexpressing primary mouse lung fibroblasts using iodoTMT reagents. Three replicate cultures of young, old, and old fibroblasts transfected with Slc7a11 were plated in separate dishes. Proteins from each dish were labeled sequentially with 2 different iodoTMT tags. For multiplexed mass spectrometry analysis, 3 mixes were produced by pooling labeled proteins from each experimental condition. Thus, each mix contained proteins labeled with 6 different iodoTMT tags. The detailed procedure is described in the main text.

Briefly, equal amounts of total protein from different groups labeled as described in Section 2.7 were pooled together to make 3 separate mixes, as outlined in Figure 1. Samples were digested overnight using mass spectrometry grade trypsin at 37°C with agitation. After digestion, samples were lyophilized, redissolved in 100 *μ*l of Tris-buffered saline (TBS), and incubated with 0.2 ml of anti-TMT resin beads (0.4 ml of 50% slurry) at 4°C overnight to enrich the labeled peptides. The affinity resin was washed 3 times with 200 *μ*l of TBS, followed by 3 times with 0.2 ml water, and then bound peptides were eluted 3 times with 0.2 ml elution buffer. The elutes were then desalted with the C_18_ spin column (Pierce, Rockford, IL), dried by speed vacuum, and dissolved in 1% formic acid before analyzed by LC-MS/MS. The enriched peptides were analyzed by an UltiMate 3000 nanoLC and Q Exactive HF Orbitrap MS system (Thermo Scientific). Peptides in samples were trapped on a *μ*-precolumn and then transferred to and separated on an in-house packed C_18_ analytic column (particle size 3.6 *μ*m, 100 *μ*m i.d.×135 mm) with a 120 min binary solvent gradient (0.1% formic acid and acetonitrile with 0.1% formic acid). Eluate from the column was directly ionized by a Nanospray Flex source (Thermo Fisher) and analyzed by the mass spectrometer in data-dependent acquisition (DDA) mode. The mass resolution was set to 60,000 and 15,000 for MS and MS/MS, respectively. AGC, isolation window, and off set were set to 2e5, 2 *m*/*z*, and 0.5 *m*/*z*. Acquired data were processed by Proteome Discoverer (Thermo Scientific v.1.4.1.14) with Sequest HT and Mascot (v2.5.1) search engines using the NCBI mouse protein database (2/9/2018 download). Data were searched considering two missed tryptic cleavages, dynamic modifications cysteine residues by iodoTMT 6plex tag, and methionine oxidation were applied in the search. The match tolerances were set to 20 ppm, 5 mDa, and 5 mDa for precursor, fragment, and report ions, respectively. The target-decoy PSM validator node in PD v1.4 was used to estimate the false discovery rates (FDR) for peptide identifications. Peptide and protein scoring was as described in 2.8. Relative quantification of sample dependent peptide abundances were based on reporter ion intensities used to calculate intensity ratios.

### 2.10. Ingenuity Pathway Analysis (IPA)

Redox state of a cysteine residue in a protein was defined as the ratio of its oxidized form to reduced form. Peptides containing cysteine residues that had a significant change of redox state with aging were selected. Among them, the redox states of cysteines in a subgroup of peptides were reversed by Slc7a11 overexpression. Proteins containing peptides in this subgroup were used for IPA (http://www.ingenuity.com) and bioinformatic analyses. IPA predicts networks, canonical pathways, and molecular functions associated with the input list of genes or proteins [26]. Fold-changes of the redox states of cysteines between old and young fibroblasts for the above subgroup were used as IPA input. The false discovery rate (FDR) threshold was set at 0.05.

### 2.11. Gene Ontology (GO) Enrichment and Kyoto Encyclopedia of Genes and Genomes (KEGG) Pathway Analysis

GO analysis is used for annotating genes and their products from 3 categories, including biological process (BP), molecular function (MF), and cellular component (CC) [27]. KEGG (http://www.genome.jp/kegg/) is a database for linking genomic information with functional information to achieve the analysis of functions of genes systematically [28]. Online Database for Annotation, Visualization, and Integrated Discovery (DAVID, https://david.ncifcrf.gov/) was used to conduct GO enrichment analysis and KEGG pathway analysis [29]. GO and KEGG terms with a *p* value < 0.05 were considered significantly enriched.

### 2.12. Protein-Protein Interaction (PPI) Network Construction

STRING database (http://www.string-db.org/) and Cytoscape 3.6.1 (https://cytoscape.org/) were used to construct the PPI network to explore functional associations between proteins [30, 31]. The minimum required interaction score was medium confidence (≥0.4).

### 2.13. Statistical Analyses

Differences in extracellular E_h_(Cys/CySS) and Slc7a11 expression were determined by 1-way ANOVA and Tukey's post hoc test, assuming that a *p* value adjusted for multiple comparisons less than 0.05 was significant. Peptides identified by mass spectrometry were considered to have a redox state that was significantly altered by age if the mean value for the 3 young replicate cultures yielded a *p* value less than 0.05 by Student's *t* test when compared to the 3 old replicate cultures. The ability of Slc7a11 overexpression to restore the redox state in old cells to that seen in young cells was assessed by comparison of the redox state of a given peptide in young cells to that in old cells overexpressing Slc7a11. A *p* value greater than 0.05 by Student's *t* test indicated that Slc7a11 overexpression normalized the redox state of that protein.

## 3. Results

### 3.1. Slc7a11 Overexpression Corrects Extracellular E_h_(Cys/CySS) in Old Fibroblasts

**Figure 2:**
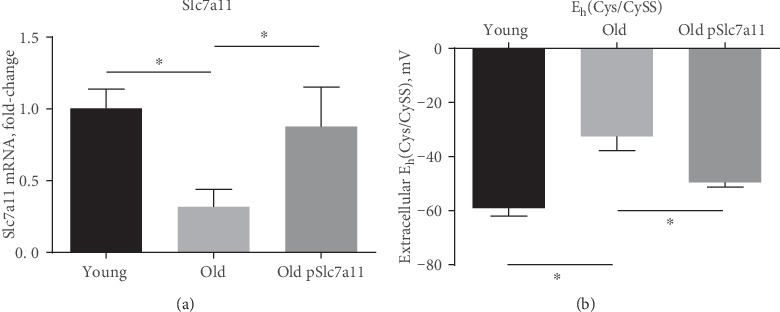
Overexpression of Slc7a11 restored age-dependent oxidation of extracellular E_h_(Cys/CySS) in old fibroblasts. (a) Slc7a11 mRNA expression and (b) extracellular E_h_(Cys/CySS) were measured as described in materials and methods. Primary lung fibroblasts from one young and one old mouse were isolated, and old fibroblasts were transfected with Slc7a11-overexpressing plasmid (pSlc7a11). For each experimental condition, 3 replicate wells of a 6-well plate were seeded with fibroblasts, and 24 h later, the media were changed to 0 mV redox media for 4 h. Data are expressed as mean ± standard deviation of 3 independent replicates. ^∗^ indicates *p* < 0.05 by 1-way ANOVA with Tukey's post hoc test adjusted for multiple comparisons.

Our goal was to explore how aging affects redox states of intracellular cysteine residues of proteins and to examine whether Slc7a11 overexpression in old cells reverses some, if not all, of those changes. Consistent with our previous studies [4, 8], primary lung fibroblasts from old mice showed lower Slc7a11 expression (*p* = 0.029) and more oxidized extracellular E_h_(Cys/CySS) (*p* = 0.0003) compared to fibroblasts harvested from young mice (Figure 2). Genetic overexpression of Slc7a11 by plasmid in old fibroblasts successfully restored Slc7a11 expression and resulted in the reduction of extracellular E_h_(Cys/CySS) close to the levels seen in young fibroblasts (Figure 2). Thus, even though the media was changed to 0 mV media 4 hours before these measurements, each culture had already conditioned its media to the preferred E_h_(Cys/CySS). Due to lot-to-lot variability in the specificity of commercially available antibodies [32], we were not able to determine whether the restoration of mRNA levels also correspondingly increased protein levels, but our earlier studies showed a close correlation between Slc7a11 mRNA levels and activity of Slc7a11 in these cells.

### 3.2. Identification of Intracellular Protein Cysteines whose Redox States Changed with Aging but Were Restored via Slc7a11 Overexpression

**Figure 3:**
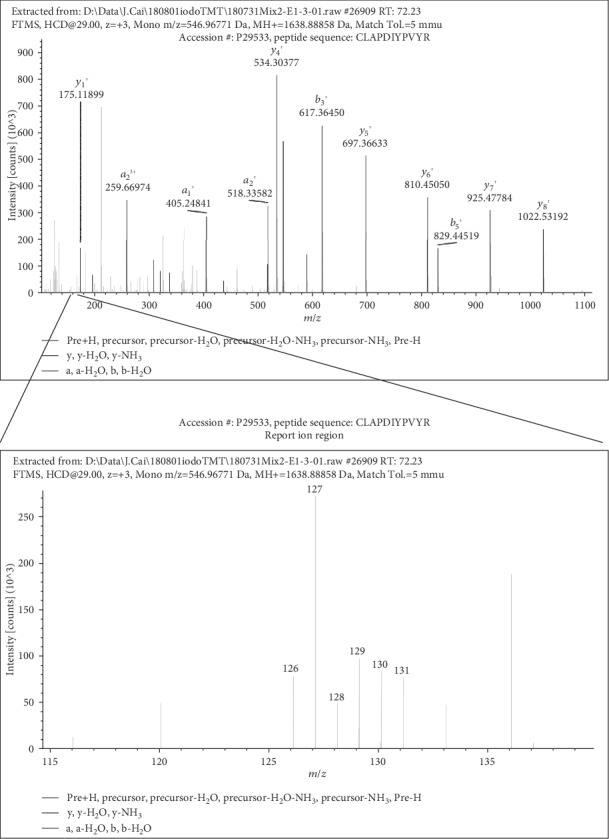
Representative mass spectrum for determining Cys redox state. Peptides in the mixed samples were ionized and separated by LC-MS/MS. Specific Cys-containing peptides were selected after the first mass spectrometry based on the mass to charge ratio. Those peptides were further fragmented to smaller ions and detected by the second mass spectrometry as shown in the upper panel. The region containing peaks of the reporter ions of the six iodoTMT labels (*m*/*z* 126-131) is shown in the lower panel. The peak intensity ratios of 129/128, 131/130, or 127/126 were defined as redox states for young, old, or old Slc7a11-overexpressing primary mouse lung fibroblasts, respectively.

After collecting the media for measuring extracellular E_h_(Cys/CySS), the 3 replicate cultures of young, old, and old fibroblasts transfected with Slc7a11 were processed for intracellular cysteine redox proteomic analysis. Three different iodoTMT labels were used to label biologically reduced cysteine residues of proteins in young, old, and old Slc7a11-overexpressing fibroblasts (Figure 1). Next, TCEP was used to reduce reversibly oxidized cysteines, including disulfides, sulfenic acids, S-glutathionylation, and S-nitrosylation. Then, 3 different tags were used to label those originally oxidized cysteines. Following digestion and enrichment via iodoTMT-affinity resin, peptides were analyzed via LC-MS/MS. A representative MS/MS spectrum is shown to demonstrate the identification of peptides and quantification of oxidized and reduced forms from reporter ions (Figure 3).

**Figure 4:**
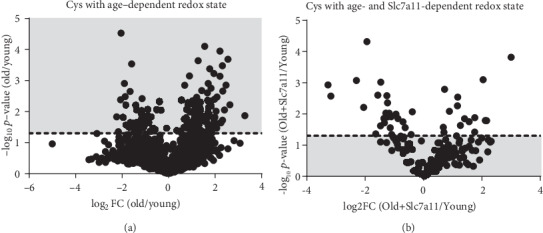
Volcano plots showing peptides with age-dependent and Slc7a11-reversible Cys redox states. Each dot represents one Cys-containing peptide. (a) Age-dependent peptide redox states are depicted as the fold-change (FC) in old relative to young cells on the *x*-axis and *p* value on the *y*-axis. The 151 peptides above the dashed line (shaded area) were considered to contain cysteines whose redox states changed significantly in response to aging (age-dependent Cys redox state). (b) The effects of Slc7a11 overexpression on the age-dependent peptides from panel A are shown. Old cells transfected with Slc7a11 were compared to young cells. The peptides containing age-dependent Cys redox states in panel A were selected and plotted. Peptides below the dashed line (shaded area) were considered to contain Slc7a11-reversible Cys redox states. Altogether, 104 proteins contained 116 cysteines whose redox states were reversed by Slc7a11 overexpression. Three replicates of young, old, and old Slc7a11-overexpressing fibroblasts were used to calculate the *p* values.

Over one thousand peptides were identified in the iodoTMT experiments. To facilitate quantitative comparisons, the redox state of a specific cysteine residue was represented by the ratio of its oxidized portion to its reduced portion (redox state of cysteine = oxidized portion/reduced portion). To determine how the redox states of individual cysteines differed between old and young cells, a volcano plot representing statistical significance on the *y*-axis and fold-change of cysteine redox states on the *x*-axis was produced (Figure 4(a)). For each cysteine residue, change of redox state with aging was represented by fold-change (FC) of redox state in old cells relative to young cells (Old vs. Young FC = redox state of cysteine in old cells/redox state of cysteine in young cells) (Figure 4(a)). Among all cysteines with detectable redox states, 12.6% (162/1282) peptides contained differentially oxidized cysteines with aging (above the dashed line representing *p* value cutoff of 0.05) (Figure 4(a)). Of those, 69% (112/162) were more oxidized in the old cells than in the young cells (above the dashed line and log_2_FC(old/young) > 0), while 31% (50/162) were more reduced with aging (above the dashed line and log_2_FC(old/young) < 0) (Figure 4(a)).

The 162 peptides identified to be more oxidized or more reduced in the old cells than in the young cells that belonged to 151 proteins and were selected to further explore whether their redox states were reversible via the Slc7a11 overexpression. The reversibility of age-dependent changes of redox state was assessed by comparing the redox states of the age-dependent peptides identified in Figure 4(a) in old Slc7a11-overexpressing cells relative to young cells (old Slc7a11 overexpression vs. young FC = redox state of cysteine in old Slc7a11-overexpressing cells/redox state of cysteine in young cells) (Figure 4(b)). Noticeably, 71.6% (116/162) of age-dependent changes of cysteine redox states were reversible via Slc7a11 overexpression (below the dashed line) (Figure 4(b)). The majority of Slc7a11-dependent redox-sensitive cysteines (93 of 116, or 80%) were more oxidized in old cells, but a substantial number (23 of 116, or 20%) were more reduced in old cells. The 116 Slc7a11-dependent cysteine-containing peptides belonged to 104 distinct proteins, with one protein containing both oxidized and reduced cysteines. Those 104 proteins constituted the majority (104 of 151, or 69%) of the age-dependent redox-modified proteins (Supplementary Table 1). IPA analysis of proteins whose redox state changes with Slc7a11 overexpression in old fibroblasts regardless of their redox states in young fibroblasts is provided in the Supplementary Material (Supplementary Table 2).

The above observations indicated that the decreased expression of Slc7a11 with aging might significantly contribute to the age-dependent global changes observed in the redox states of the intracellular cysteine proteome in primary mouse lung fibroblasts. We focused on the 104 proteins that change with age and are reversible upon the restoration of the Slc7a11 expression to further explore their potential biological functions using bioinformatics analyses.

### 3.3. Ingenuity Pathway Analysis (IPA), Gene Ontology (GO) and Pathway Enrichment Analyses, and Protein-Protein Interaction (PPI) Network Analysis of Proteins That Exhibit Age-Dependent Changes in Redox State That Are Reversed upon Overexpression of Slc7a11

IPA showed that the most significantly enriched pathways related to Slc7a11-dependent redox-sensitive proteins were eukaryotic translation initiation factor 2 (EIF2) signaling, actin cytoskeleton and integrin-linked kinase (ILK) signaling, and protein ubiquitination pathway, which represented protein synthesis, cellular structure and communication, and protein ubiquitin-proteasome-mediated degradation, respectively (Table 1). Three actin-associated cytoskeleton proteins, TLN1, FLNB, and PPP2CA, contained multiple redox-sensitive cysteines, indicating that actin dynamics and communication between the cell membrane and intracellular cytoskeleton were prone to age-related redox regulation and, importantly, that Slc7a11 overexpression reversed these effects of aging (Table 1). Interestingly, not all cysteine residues became more oxidized with aging. On the contrary, some became more reduced (Table 1), indicating that although aging promoted the overall oxidation of the redox environment, the redox states of distinct components of the intracellular cysteine proteome were differentially regulated instead of simply being oxidized. Slc7a11 overexpression not only reduced age-dependent oxidation of proteins, but also oxidized age-dependent reduction of certain proteins, suggesting that Slc7a11 influences the cysteine proteome indirectly via restoring the global intracellular redox environment rather than interacting directly with individual redox-sensitive proteins.

**Figure 5:**
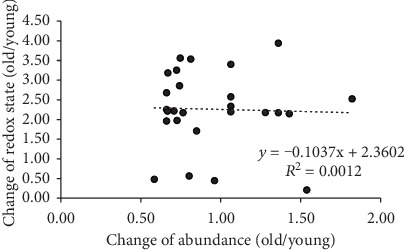
Changes in the redox state do not correlate with changes in protein abundance. The abundance and redox state data from Table 1 were plotted for each protein for which both values were determined. Linear regression analysis showed no correlation between the two values.

The function of any given protein is determined not only by its redox state, but also by other factors, including abundance level. In a separate experiment, the effects of aging on protein abundance were assessed by two-dimensional LC-MS/MS using a TMT 10plex approach as described in Methods Section 2.8. This analysis identified 5,081 unique proteins based on the 2-unique peptide acceptance criteria. Of these, 159 were ≥2-fold higher in old cells than in young cells, while 310 were ≥2-fold lower in old cells (not shown). However, none of the proteins found to have age-dependent, and Slc7a11-regulated redox states were among these proteins with abundance differences greater than 2-fold (Table 1). To further investigate whether there was a relationship between redox state and abundance, linear regression analysis was performed. When the change in redox state was compared to the change in abundance for individual proteins, no correlation was evident between the 2 measures (Figure 5).

GO is a collection of terms describing gene functions and the relationship between the terms. It consists of 3 aspects: *biological process*, *molecular function*, *and cellular component*. Consistent with IPA, GO annotation for *biological process* revealed that Slc7a11-dependent redox-sensitive proteins were enriched in cell adhesion, initiation, and regulation of protein translation, organization of actin cytoskeleton and proteolysis (Table 2). Other significant enriched terms related to *biological process* involved RNA processing, cellular response to stimuli (e.g., virus), and oxidation-reduction processes (Table 2). Furthermore, Slc7a11 affected the redox states of proteins with *molecular function* related to the binding of poly(A) RNA, cellular adhesion-related cadherin, actin, ATP, or other proteins (Table 3). Related to the GO *cellular component*, enriched items included extracellular exosome, cell-cell adherens junctions, and cytoplasm (Table 4). Finally, KEGG pathways were mainly enriched in carbon metabolism and amino acids biosynthesis, ribosome and proteasome, tight and adherens junctions, and focal adhesion (Table 5). IPA and GO-based biological process analyses of proteins that exhibit age-dependent changes in redox state that are not reversed upon overexpression of Slc7a11 are provided in the Supplementary Material (Supplementary Tables 3 and 4).

**Figure 6:**
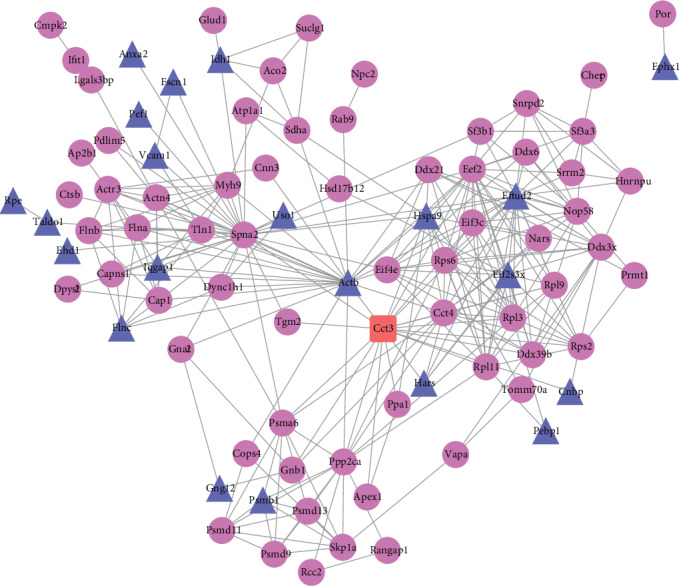
Protein-protein interaction (PPI) network showed proteins with age-dependent Slc7a11-responsive cysteines were clustered in IPA-predicted pathways. The upper left cluster was associated with cytoskeleton and cellular interaction, including actin signaling and integrin-linked kinase (ILK) signaling. The upper right cluster was associated with protein translation, including eukaryotic translation initiation factor 2 (EIF2) signaling. The lower cluster was related to protein ubiquitination and degradation. Proteins that contained age-dependent oxidized, reduced, or both oxidized and reduced cysteines were shown in round, triangular, or rectangular boxes, respectively.

PPI network demonstrates the interactions between proteins in a straightforward and visualized way. Proteins with functional connections are clustered. We identified 3 clusters from our PPI network (Figure 6). The upper left cluster was associated with cytoskeleton and cellular interaction, including actin signaling and integrin-linked kinase (ILK) signaling (Figure 6). The upper right cluster was involved in protein translation pathways, including EIF2 signaling (Figure 6). The lower cluster was related to protein ubiquitination and proteasome-mediated degradation (Figure 6). Those data were consistent with the IPA results and suggested that, by reversing the intracellular redox environment, Slc7a11 was able to block the age-dependent changes of redox states of protein cysteines in pathways of cytoskeleton dynamics and protein turnover.

## 4. Discussion

The current study confirms the role of Slc7a11 in mediating age-dependent oxidization of extracellular E_h_(Cys/CySS) and extends these findings to include the impact of changes in Slc7a11 expression on the redox states of multiple intracellular proteins. We find that proteins related to the cytoskeleton or involved in protein degradation and protein synthesis are differentially oxidized in old murine lung fibroblasts as a result of lower Slc7a11 expression. The Slc7a11 overexpression not only reduces proteins that become oxidized with aging, but also oxidizes certain proteins that are reduced with aging. The effects of age and Slc7a11 on protein redox states were not associated with the changes in protein abundance. Thus, targeting Slc7a11 for redox regulation might be a better approach than simply supplementing with antioxidants to reestablish redox homeostasis in aging and diseases.

The redox states of particular cysteines associated with actin cytoskeleton signaling and ILK signaling are age-dependent but can be restored with the Slc7a11 overexpression. Integrins mediate cell-cell and cell-matrix interactions, thereby linking extracellular matrices with the intracellular cytoskeleton. Talin and alpha-actinin, which are components of focal adhesion complexes [33, 34], are shown in our study to contain cysteines whose redox states are oxidized with aging. These changes might impact integrin-mediated signaling or mechanotransduction through alterations in cell stiffness, a process considered critical for promoting aberrant lung remodeling after injury and fibrosis [35, 36]. Others have shown that cytoskeletal proteins are oxidized with aging. Protein carbonylation is an irreversible oxidative modification that is increased in the brains of old senescence-accelerated-prone 8 (SAMP8) mice compared to the brains of young mice [37]. In particular, collapsin response mediator protein-2 (CRMP-2), which is involved in cytoskeletal remodeling, microtubule assembly and cell migration, and alpha-spectrin, which interacts with actin to form a scaffold to maintain cytoskeletal stability and flexibility, is more oxidized [38]. Thus, the actin cytoskeleton system might be a major target of age-dependent redox modification. S-glutathionylation is a form of reversible oxidation of cysteines. Actin contains cysteines with different degrees of S-glutathionylation that are flexibly deglutathionylated via glutaredoxin in response to stimuli [39]. Glutaredoxin-mediated changes of cysteine S-glutathionylation may regulate actin polymerization [40]. These findings are considered important as the actin cytoskeleton is believed to play critical roles in fibroblast differentiation, myofibroblast contraction, focal adhesion complex formation, extracellular matrix remodeling, mechanical to biochemical signal transduction, and gene transcription, which are all important biological processes proposed to be involved in tissue fibrogenesis and maladaptive wound healing [41]. In human skin fibroblasts, disassembly of actin cytoskeleton results in disruption of the TGF-*β* signaling pathway with subsequent collagen production, thus promoting skin aging [42]. These findings further support the idea that the function of the actin cytoskeleton system is redox-sensitive, and its disruption might be associated with aging-related degeneration and disease development.

Loss of proteostasis is a hallmark of aging [2]. Proteostasis (from the terms protein and homeostasis) refers to the balance among the processes of protein synthesis, folding, trafficking, and degradation. Redox modulation has been shown to affect protein folding and trafficking [43, 44]. Disruption of proteostasis can result in the deposition of protein aggregates and be harmful for cell survival [45, 46]. Protein degradation by the proteasome changes with aging and is regulated by redox modulation, especially thiol oxidation [47, 48]. Here, we identified three age-oxidized cysteines in proteasome 26S subunits and one age-reduced cysteine in heat shock protein 70 (HSP70), providing further evidence for the idea that the 26S proteasome is vulnerable to redox regulation, and that its chaperone HSP70 responds to redox regulation [49, 50]. The implications of age-dependent changes of protein redox states in the promotion of aging-associated diseases require further exploration. However, our studies suggest that they are important and are in line with other observations. For instance, in the hippocampi of patients with Alzheimer disease (AD), peptidyl-prolyl cis-trans isomerase 1 (Pin 1), which catalyzes the isomerization of the neuronal cytoskeleton protein tau, becomes more oxidized [51]. Ubiquitin carboxyl terminal hydrolase L-1 (UCHL-1) is involved in proteasome-mediated protein degradation, and its oxidation in AD disrupts the ubiquitination/de-ubiquitination balance and results in the accumulation of dysfunctional proteins [51]. Furthermore, proteins in the cell structural and proteasomal pathways are oxidatively modified in Parkinson's disease [52].

Others have identified cysteine redox modifications that affect proteostasis. For example, S-glutathionylation of multiple cysteine residues results in increased accessibility of proteasomal active center and increased proteolytic activity [53]. We identified several other novel reversible redox-sensitive cysteines that might also influence proteasomal activity. Whether proteosomal activity is increased or decreased depends on both the location and specific form of the redox modification being evaluated, thereby requiring experimental exploration in the future. Our data show that aging is associated with lower caspase-like and chymotrypsin-like proteasomal activity, but that only caspase-like activity could be restored upon the upregulation of Slc7a11 by sulforaphane (Supplementary Figure 1). Intriguingly, the *β*1 subunit of the 20S core (Psmb1) was found to be more reduced in old cells (Table 1), and it is this subunit that confers caspase-like activity to the proteasome [54–56]. Our observations suggest that both cytoskeleton and proteasomal pathways are susceptible to age-related redox regulation, and that redox modulations of cysteines might be an important posttranslational mechanism responsible for age-dependent change of protein functions in those pathways.

Protein synthesis was also found to be disrupted with aging and modified by Slc7a11-associated change of the redox environment. Compared to relatively well-studied age-dependent protein degradation, how protein synthesis is altered with aging remains to be explored. Our study found that aging was associated with changes of redox states of cysteines in EIF2 signaling, including EIF4E, RPS6, and RPL9. These three proteins regulate protein synthesis in aging [57]. The concentration and activity of eukaryotic translation initiation factors (eIFs) and the abundance of ribosomes are decreased with aging, resulting in an overall decrease in protein synthesis [58]. These and the identification of several other age-dependent cysteines in other parts of eIFs and ribosomes suggest that cysteine redox modifications mediate age-dependent defects detected in the protein synthesis machinery. The function of eIF2, for instance, is to load methionyl-tRNA to the 40S ribosomal subunit, which is the first step of 43S preinitiation complex assembly [59]. The function of eIF4E is to bind with the 5′ cap of mRNAs and mediate recruitment and attachment of the 43S preinitiation complex [60]. Those two steps determine the rate of protein synthesis. Phosphorylation of those initiation factors and their regulatory factors has been shown to control protein synthesis [61, 62]. Similar to phosphorylation, redox modifications might represent another posttranslational mechanism capable of affecting the efficacy of protein synthesis. This idea is further supported by a recent study that revealed several H_2_O_2_-sensitive thiols in proteins involved in the general translation machinery, and the subsequent attenuation of protein synthesis via such redox modulation [63]. More studies are needed to further elucidate this redox control of protein synthesis pathways.

Perhaps the most important finding of our study is that the Slc7a11 overexpression may restore protein redox microenvironments. Accordingly, one could predict that alterations in the Slc7a11 expression or activity would lead to diseased states. Consistent with this idea, others reported that the increase of Slc7a11 was part of the signatures of senescence inflammatory responses in intestinal epithelial cells [64]. In peripheral white blood cells from schizophrenia patients, the Slc7a11 expression was lower compared to those from healthy donors [65]. In tissues of nonsmall cell lung cancer, the Slc7a11 expression was higher [66]. In glioma cells, the C-terminus of EGFR directly interacts with the central part of Slc7a11 and stabilizes Slc7a11 cell surface expression [67], thereby suggesting a role in oncogenic signaling. CD44 variant directly interacts with Slc7a11 in pulmonary artery endothelial cells suggesting a role in vascular remodeling [68]. Those interactions further complicate the potential biological roles of Slc7a11. It is unclear whether and how Slc7a11 expression changes in different cell types and whether such changes affect the progression of aging or disease development differently. Although the upstream or downstream pathways of Slc7a11 might be different, the universal underlying mechanism is usually associated with Slc7a11-related change of redox environment, suggesting that redox modulation might play a central role in controlling multiple biological processes and signaling pathways.

Slc7a11 provides cells with Cys to support GSH synthesis. Therefore, one of the functions of the Slc7a11 activity may be to support the reduction of oxidized proteins by providing an essential co-factor for glutaredoxins [69]. Glutaredoxins complement thioredoxins to reduce proteins oxidized by glutathionylation, sulfenic acid or disulfide formation, or S-nitrosylation [70–74]. The link between Slc7a11 activity and thioredoxin-dependent protein reduction is not as direct as it is for glutathione-dependent processes, but an increase in GSH synthesis may decrease the overall burden on the thioredoxin system. Our results showed that while most cysteines became more oxidized in lung fibroblasts from aged mice, some became more reduced, and Slc7a11 overexpression reversed redox states of both oxidized and reduced cysteines, supporting the idea that Slc7a11 manipulation targets intracellular redox signaling pathways rather than simply decreases overall oxidative stress. These findings are consistent with the concept that physiological levels of oxidants are necessary for the proper functioning of biological systems and contribute to a condition of oxidative eustress [75] wherein cysteine residues on key regulatory proteins are maintained at optimal redox states. In this context, aging may represent a shift toward a condition of oxidative distress in which certain proteins are no longer in their ideal redox state [76]. Our findings provide insight for novel ways to combat redox changes associated with aging and age-related aberrant wound healing.

Limitations of the present study include the use of cells isolated from the lungs of only one female mouse of each age and incomplete coverage of the redox proteome. Sex differences in redox regulation and glutathione metabolism have been observed [77, 78], and additional studies will be needed to ascertain whether there are sex differences in Slc7a11 expression and the redox proteome with aging. The present studies used only technical replicates of cells isolated from one young and one old mouse. It will be important to confirm these findings using cells from additional mice (i.e., biological replicates). Biological replicates were used in the validation experiments (Supplementary Materials), showing that the predicted changes in proteasomal activity were indeed consistent between mice of a given age. It would also be of interest to repeat these studies in cells from other tissues and other species to determine if the findings are generally applicable. The interpretation of the results of Slc7a11 overexpression in old cells is somewhat complicated by the absence of electroporation controls in the present study. Our previous studies showed that the electroporation itself had no effect on either the expression of Slc7a11 or the extracellular E_h_(Cys/CySS) [8]. However, the redox proteome may be more sensitive to electroporation than these other parameters. The proteins and pathways identified here derive from an analysis of proteins digested with trypsin. Therefore, some protein cysteines were missed because they reside within peptides that were either too large or too small for detection or identification by tandem mass spectrometry.

## 5. Conclusions

Aging is associated with the disruption of the intracellular redox environment represented by changes in the redox states of cysteines in fundamental pathways including protein turnover and cytoskeleton organization. Slc7a11 is a critical factor not only for regulating the extracellular redox environment, but also for controlling the intracellular redox environment. Targeting Slc7a11 can reverse age-dependent effects on intracellular redox signaling pathways and represents a novel approach to prevent and treat age-related diseases.

## Figures and Tables

**Table 1:** Ingenuity Pathway Analysis (IPA) that indicated proteins whose redox states were restored by the Slc7a11 overexpression were enriched in pathways of protein turnover and cytoskeleton signaling. The top 3 most significant enriched pathways were protein synthesis pathway, protein ubiquitin-proteasome-mediated degradation pathway, and actin cytoskeleton signaling/integrin-linked kinase (ILK) signaling. Some proteins contained more than 1 redox-sensitive Cys residue. Some Cys residues became more oxidized with aging while others became more reduced.

Related IPA pathway	Change of redox state	Protein	Description	Redox-sensitive Cys	Abundance (old/young)	Redox state (old/young)
Protein synthesis pathway (EIF2 signaling)	Oxidized	EIF3C	Eukaryotic translation initiation factor 3 subunit C	GTTEEI**C**QIYLR	0.74	2.86
		EIF4E	Eukaryotic translation initiation factor 4E	IAIWTTE**C**ENR	0.67	2.22
		RPL3	Ribosomal protein L3	VA**C**IGAWHPAR	0.81	3.54
		RPL9	Ribosomal protein L9	TGVA**C**SVSQAQK	N/A	4.76
		RPL11	Ribosomal protein L11	IAVH**C**TVR	0.71	2.22
		RPS2	Ribosomal protein S2	G**C**TATLGNFAK	0.76	2.17
		RPS6	Ribosomal protein S6	LNISFPATG**C**QK	0.67	3.19
	Reduced	ACTB	Actin beta	L**C**YVALDFEQEMATAASSSSLEK	N/A	0.31
		EIF2S3	Eukaryotic translation initiation factor 2 subunit gamma	IVLTNPV**C**TEVGEK	0.96	0.45

Actin cytoskeleton signaling/ILK signaling	Oxidized	ACTN4	Actinin alpha 4	A**C**LISLGYDVENDR	1.43	2.15
		ACTR3	ARP3 actin-related protein 3 homolog	YSYV**C**PDLVK	1.28	2.18
		FLNA	Filamin A	SNFTVD**C**SK	N/A	2.45
				**C**SGPGLSPGMVR	N/A	3.86
		MYH9	Myosin heavy chain 9	KQELEEI**C**HDLEAR	1.82	2.53
		TLN1	Talin 1	QVAASTAQLLVA**C**K	1.06	2.20
				**C**VS**C**LPGQR	1.06	2.34
				VVAPTISSPV**C**QEQLVEAGR	1.06	3.40
				AGALQ**C**SPSDVYTK	1.06	2.58
		FLNB	Filamin B	IAGPGLSS**C**VR	1.36	2.17
				A**C**IPQSFTVDSSK	1.36	3.94
		PPP2CA	Protein phosphatase 2 catalytic subunit alpha	AHQLVMEGYNW**C**HDR	0.66	2.26
				QITQVYGFYDE**C**LR	0.66	2.68
	Reduced	ACTB	Actin beta	L**C**YVALDFEQEMATAASSSSLEK	N/A	0.31
		GNG12	G protein subunit gamma 12	ASADLMSY**C**EEHAR	N/A	0.94
		IQGAP1	IQ motif containing GTPase activating protein 1	FFQVA**C**DVPELQDK	1.54	0.21
		FLNC	Filamin C	DGS**C**GVSYVVQEPGDYEVSIK	N/A	0.22

Protein degradation pathway (proteasome-mediated)	Oxidized	PSMA6	Proteasome subunit alpha 6	YGYEIPVDML**C**K	0.85	1.71
		PSMD9	Proteasome 26S subunit, non-ATPase 9	GIGMNEPLVD**C**EGYPR	0.73	1.98
		PSMD11	Proteasome 26S subunit, non-ATPase 11	TTANAIY**C**PPK	0.72	3.26
		PSMD13	Proteasome 26S subunit, non-ATPase 13	SSDEAVIL**C**K	0.75	3.56
		SKP1	S-phase kinase-associated protein 1	GLLDVT**C**K	0.66	1.96
	Reduced	HSPA9	Heat shock protein family A (Hsp70) member 9	**C**ELSSSVQTDINLPYLTMDASGPK	0.58	0.48
		PSMB1	Proteasome subunit beta 1	I**C**IVTK	0.80	0.57
		USO1	USO1 vesicle transport factor	SQL**C**SQSLEITR	N/A	0.39

**Table 2:** GO-based biological process analysis of proteins whose redox states were rescued by Slc7a11 overexpression in old fibroblasts. Fisher's exact test *p* values for all the items shown were lower than 0.05.

Term	Count	*p* value	-log10 (*p* value)
Cell-cell adhesion	11	<0.001	7.197
Translation	11	<0.001	4.274
RNA splicing	8	<0.001	3.526
Tricarboxylic acid cycle	4	<0.001	3.319
Platelet aggregation	4	0.001	2.971
mRNA processing	8	0.002	2.786
RNA secondary structure unwinding	4	0.002	2.757
Translational initiation	4	0.003	2.529
Cellular response to peptide hormone stimulus	3	0.007	2.147
Formation of translation preinitiation complex	3	0.008	2.112
Establishment or maintenance of cell polarity	3	0.009	2.047
Response to virus	4	0.010	1.994
Pentose-phosphate shunt, nonoxidative branch	2	0.026	1.581
Oxidation-reduction process	9	0.027	1.564
Actin cytoskeleton reorganization	3	0.031	1.507
Isocitrate metabolic process	2	0.031	1.503
Proteolysis involved in cellular protein catabolic process	3	0.037	1.433
Positive regulation of translation	3	0.037	1.433
Neuron projection development	4	0.038	1.422
Actin cytoskeleton organization	4	0.040	1.399

**Table 3:** GO-based molecular function analysis of proteins whose redox states were rescued by Slc7a11 overexpression in old fibroblasts. Fisher's exact test *p* values for all the items shown were lower than 0.05.

Term	Count	*p* value	-log10 (*p* value)
Poly(A) RNA binding	39	<0.001	20.191
Cadherin binding involved in cell-cell adhesion	20	<0.001	15.060
Enzyme binding	13	<0.001	5.841
Actin binding	12	<0.001	5.558
Actin filament binding	8	<0.001	5.054
RNA binding	16	<0.001	4.598
Protein complex binding	11	<0.001	4.521
Protein binding	42	<0.001	4.517
Nucleotide binding	25	<0.001	3.935
Protein domain specific binding	9	<0.001	3.682
GTP binding	9	0.001	2.878
ATP binding	19	0.001	2.826
Protein kinase binding	9	0.003	2.540
ATP-dependent RNA helicase activity	4	0.005	2.284
GTPase activity	6	0.006	2.206
Oxidoreductase activity	10	0.006	2.191
ADP binding	3	0.017	1.768
Rac GTPase binding	3	0.022	1.662
Endopeptidase activity	4	0.025	1.610
Nucleoside binding	2	0.027	1.561

**Table 4:** GO-based cellular component analysis of proteins whose redox states were rescued by Slc7a11 overexpression in old fibroblasts. Fisher's exact test *p* values for all the items shown were lower than 0.05.

Term	Count	*p* value	-log10 (*p* value)
Extracellular exosome	57	<0.001	23.688
Cell-cell adherens junction	20	<0.001	14.949
Cytoplasm	72	<0.001	14.704
Focal adhesion	16	<0.001	9.092
Myelin sheath	12	<0.001	8.558
Extracellular matrix	13	<0.001	7.639
Cytosol	28	<0.001	7.085
Intracellular ribonucleoprotein complex	12	<0.001	6.291
Spliceosomal complex	8	<0.001	5.304
Actin cytoskeleton	9	<0.001	5.162
Cytoplasmic ribonucleoprotein granule	5	<0.001	4.983
Cortical cytoskeleton	5	<0.001	4.801
Stress fiber	6	<0.001	4.700
Cytoskeleton	18	<0.001	4.543
Perinuclear region of cytoplasm	14	<0.001	4.457
Protein complex	13	<0.001	4.216
Nucleus	49	<0.001	4.123
Extracellular vesicle	5	<0.001	4.002
Catalytic step 2 spliceosome	6	<0.001	3.947
Proteasome complex	5	<0.001	3.498

**Table 5:** KEGG pathway analysis of proteins whose redox states were rescued by Slc7a11 overexpression in old fibroblasts. Fisher's exact test *p* values for all the items shown were lower than 0.05.

Term	Count	*p* value	-log10 (*p* value)
Carbon metabolism	8	<0.001	4.183
Spliceosome	7	0.001	2.972
Tight junction	7	0.001	2.873
Citrate cycle (TCA cycle)	4	0.003	2.569
Salmonella infection	5	0.005	2.317
Proteasome	4	0.007	2.147
Biosynthesis of antibiotics	7	0.011	1.955
Adherens junction	4	0.025	1.597
Biosynthesis of amino acids	4	0.031	1.507
Proteoglycans in cancer	6	0.033	1.484
Focal adhesion	6	0.035	1.453
Ribosome	5	0.039	1.414

## Data Availability

All data used to support the findings of this study are included within the article and the supplementary information file.

## Supplementary Materials

Supplementary Materials Supplementary Figure 1. Caspase-like (top) and chymotrypsin-like (bottom) proteasomal activity. Fibroblasts were isolated from the lungs of 2 young (#1 and #2) and 2 old (#1 and #2) female C57BL/6J mice. Caspase-like and chymotrypsin-like proteasomal activities were measured before and after the induction of Slc7a11 activity with sulforaphane as described in Supplementary Methods. Both types of proteasomal activity were lower in old cells, but sulforaphane treatment restored only caspase-like proteasomal activity. Fibroblasts from 2 different young mice had similar caspase- and chymotrypsin-like activities. Fibroblasts from both old mice had lower activities than either young mouse, but one biological replicate had even lower chymotrypsin-like activity than the other. Six wells were plated for each condition. ^∗^–*p* < 0.05 by 2- way ANOVA and Tukey's post hoc test. Supplementary Table 1A: proteins with cysteines whose redox state became more oxidized with aging and restored by Slc7a11 overexpression. Supplementary Table 1B: proteins with cysteines whose redox state became more reduced with aging and restored by Slc7a11 overexpression. Supplementary Table 2: IPA analysis of proteins whose redox states were changed by Slc7a11 overexpression in old fibroblasts. Fisher's exact test *p* values for all the items shown were less than 0.05. Supplementary Table 3: IPA analysis of age-dependent proteins whose redox states were not rescued by Slc7a11 overexpression in old fibroblasts. Fisher's exact test *p* values for all the items shown were less than 0.05. Supplementary Table 4: GO-based biological process analysis of proteins whose redox states were not rescued by Slc7a11 overexpression in old fibroblasts. Fisher's exact test *p* values for all the items shown were less than 0.05.
