# Supplementary material for: Redox States of Protein Cysteines in Pathways of Protein Turnover and Cytoskeleton Dynamics Are Changed with Aging and Reversed by Slc7a11 Restoration in Mouse Lung Fibroblasts
Source: Oxid Med Cell Longev. 2020 Jun 7;2020:2468986. doi: 10.1155/2020/2468986 (PMC7298344; doi:10.1155/2020/2468986)
Supplement: Supplementary Materials — Supplementary Figure 1. Caspase-like (top) and chymotrypsin-like (bottom) proteasomal activity. Fibroblasts were isolated from the lungs of 2 young (#1 and #2) and 2 old (#1 and #2) female C57BL/6J mice. Caspase-like and chymotrypsin-like proteasomal activities were measured before and after the induction of Slc7a11 activity with sulforaphane as described in Supplementary Methods. Both types of proteasomal activity were lower in old cells, but sulforaphane treatment restored only caspase-like proteasomal activity. Fibroblasts from 2 different young mice had similar caspase- and chymotrypsin-like activities. Fibroblasts from both old mice had lower activities than either young mouse, but one biological replicate had even lower chymotrypsin-like activity than the other. Six wells were plated for each condition. ∗–p < 0.05 by 2- way ANOVA and Tukey's post hoc test. Supplementary Table 1A: proteins with cysteines whose redox state became more oxidized with aging and restored by Slc7a11 overexpression. Supplementary Table 1B: proteins with cysteines whose redox state became more reduced with aging and restored by Slc7a11 overexpression. Supplementary Table 2: IPA analysis of proteins whose redox states were changed by Slc7a11 overexpression in old fibroblasts. Fisher's exact test p values for all the items shown were less than 0.05. Supplementary Table 3: IPA analysis of age-dependent proteins whose redox states were not rescued by Slc7a11 overexpression in old fibroblasts. Fisher's exact test p values for all the items shown were less than 0.05. Supplementary Table 4: GO-based biological process analysis of proteins whose redox states were not rescued by Slc7a11 overexpression in old fibroblasts. Fisher's exact test p values for all the items shown were less than 0.05. [file 2468986.f1.pdf]

Supplemental Materials

Redox states of protein cysteines in pathways of protein turnover and cytoskeleton dynamics are changed with aging and reversed by Slc7a11 restoration in mouse lung fibroblasts

Yuxuan Zheng,[1] Michael L. Merchant,[1,2] Tom J. Burke,[3] Jeffrey D. Ritzenthaler,[4] Ming Li,[2] Adam E. Gaweda,[2] Frederick W. Benz,[1] Jesse Roman,[4] Walter H. Watson[1,3]

[1] Department of Pharmacology and Toxicology, University of Louisville School of Medicine, Louisville, KY 40202, USA

[2] Department of Medicine, Division of Nephrology and Hypertension, University of Louisville School of Medicine, Louisville, KY 40202, USA

[3] Department of Medicine, Division of Gastroenterology, Hepatology and Nutrition, University of Louisville School of Medicine, Louisville 40202, KY, USA

[4] Department of Medicine, Division of Pulmonary, Allergy and Critical Care Medicine, Sidney Kimmel Medical College and Jane and Leonard Korman Respiratory Institute, Thomas Jefferson University, Philadelphia 19107, PA, USA

Correspondence should be addressed to Walter H. Watson; bert.watson@louisville.edu

# Emails

Yuxuan Zheng
yuxuan.zheng@louisville.edu

Michael L. Merchant
michael.merchant@louisville.edu

Tom J. Burke
tom.burke@louisville.edu

Jeffrey D. Ritzenthaler
jeffrey.ritzenthaler@jefferson.edu

Ming Li
ming.li@louisville.edu

Adam E. Gaweda
adam.gaweda@louisville.edu

Frederick W. Benz
frederick.benz@louisville.edu

Jesse Roman
jesse.roman@jefferson.edu

Walter H. Watson
bert.watson@louisville.edu

Supplemental Methods

Proteasomal activity assay. Proteasomal activity was measured using the Proteasome-Glo Chymotrypsin-Like and Caspase-Like Cell-Based Assays from Promega (#G8660, #G8860, Madison, WI) according to the manufacturer's instructions. Briefly, primary lung fibroblasts (15,000 cells/well) from young or old female C57BL/6 mice were plated onto a 96 well (white walled) tissue culture plate and incubated in DMEM supplemented with 10% fetal bovine serum (FBS) and 1% antibiotic-antimycotic solution in a humidified incubator with 5% CO2. After attachment, cells were treated with or without the addition of 5 µM sulforaphane for 6 hours. Afterwards, some fibroblasts were incubated in the presence of a proteasome inhibitor lactacystin (10µM, Cayman Chemical, Ann Arbor, MI) for 2 hours to identify nonspecific protease activity not related to the proteasome. The Proteasome-Glo Cell-Based Reagent (100µl) was then added to each sample and mixed for 2 min at 700 rpm using a plate shaker. Samples were incubated at room temperature for 10 minutes prior to the measurement of luminescence using a Luminoskan Ascent Luminometer (Labsystems, Helsinki, Finland). Samples were normalized to total protein measured using protein assay dye (Bio-Rad, Hercules, CA) and values were reported as relative luciferase units.

Supplemental Figure 1 shows proteasomal activity is lower in old cells, and sulforaphane treatment restores caspase-like, but not chymotrypsin-like, proteasomal activity. This figure is related to Table 1 and described in the 4th paragraph of the Discussion section.

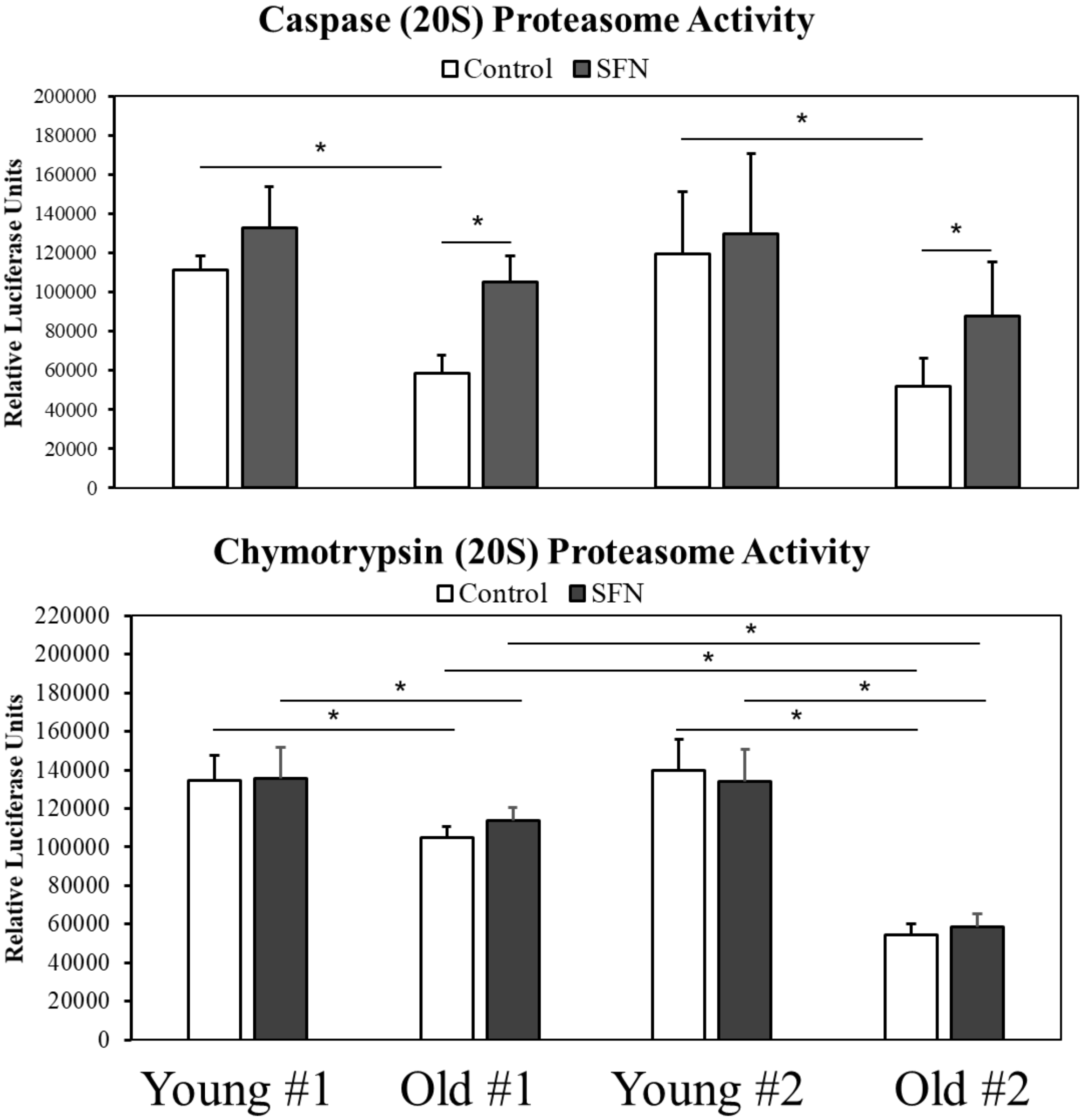

SUPPLEMENTAL FIGURE 1: Caspase-like (top) and chymotrypsin-like (bottom) proteasomal activity. Fibroblasts were isolated from the lungs of 2 Young (#1 and #2) and 2 Old (#1 and #2) female C57BL/6J mice. Caspase-like and chymotrypsin-like proteasomal activity were measured before and after induction of Slc7a11 activity with sulforaphane as described in Supplemental Methods. Both types of proteasomal activity were lower in old cells, but sulforaphane treatment restored only caspase-like proteasomal activity. Fibroblasts from 2 different young mice had similar caspase- and chymotrypsin-like activities. Fibroblasts from both old mice had lower activities than either young mouse, but one biological replicate had even lower chymotrypsin-like activity than the other. Six wells were plated for each condition. * - $p<0.05$ by 2-way ANOVA and Tukey's post-hoc test.

Supplemental Table 1 lists the total 104 proteins with age-dependent Slc7a11-responsive cysteine residues. Those proteins contain at least one cyteine whose redox state becomes more oxidized or reduced with aging and is reversible by Slc7a11 overexpression. This table is related to Figure 4(b) and Table 1 and described in the Results section 3.2. The proteins in this table are used as input for Ingenuity Pathway Analysis.

SUPPLEMENTAL TABLE 1A: Proteins with cysteines whose redox state became more oxidized with aging and restored by Slc7a11 overexpression

Sequence	Protein	Description	Protein Group Accessions	Modifications
CTTDHISAAGPWLK	Aco2	Aconitate hydratase, mitochondrial OS=Mus musculus OX=10090 GN=Aco2 PE=1 SV=1 - [ACON_MOUSE]	Q99KI0	C1(iodoTMT6plex)
ACLISLGYDVENDR	Actn4	Alpha-actinin-4 OS=Mus musculus OX=10090 GN=Actn4 PE=1 SV=1 - [ACTN4_MOUSE]	P57780	C2(iodoTMT6plex)
YSYVCPDLVK	Actr3	Actin-related protein 3 OS=Mus musculus OX=10090 GN=Actr3 PE=1 SV=3 - [ARP3_MOUSE]	Q99JY9	C5(iodoTMT6plex)
VCLLGCGISTGYGAAVNTAK	Adh5	Alcohol dehydrogenase class-3 OS=Mus musculus OX=10090 GN=Adh5 PE=1 SV=3 - [ADHX_MOUSE]	P28474	C2(iodoTMT6plex); C6(iodoTMT6plex)
DCEDPNPLIR	Ap2b1	AP-2 complex subunit beta OS=Mus musculus OX=10090 GN=Ap2b1 PE=1 SV=1 - [AP2B1_MOUSE]	Q9DBG3	C2(iodoTMT6plex)
EEAPDILCLQETK	Apex1	DNA-(apurinic or apyrimidinic site) lyase OS=Mus musculus OX=10090 GN=Apex1 PE=1 SV=2 - [APEX1_MOUSE]	P28352	C8(iodoTMT6plex)
NIAFFSTNCVEGTAR	Atp1a1	Sodium/potassium-transporting ATPase subunit alpha-1 OS=Mus musculus OX=10090 GN=Atp1a1 PE=1 SV=1 - [AT1A1_MOUSE]	Q8VDN2	C9(iodoTMT6plex)
CGLQFLGNVASR	Atxn10	Ataxin-10 OS=Mus musculus OX=10090 GN=Atxn10 PE=1 SV=2 - [ATX10_MOUSE]	P28658	C1(iodoTMT6plex)
INSITVDNCK	Cap1	Adenylyl cyclase-associated protein 1 OS=Mus musculus OX=10090 GN=Cap1 PE=1 SV=4 - [CAP1_MOUSE]	P40124	C9(iodoTMT6plex)
TDGFGIDTCR	Capns1	Calpain small subunit 1 OS=Mus musculus OX=10090 GN=Capns1 PE=1 SV=1 - [CPNS1_MOUSE]	O88456	C9(iodoTMT6plex)
CAQAQTGIDLSTCTK	Ccar2	Cell cycle and apoptosis regulator protein 2 OS=Mus musculus OX=10090 GN=Ccar2 PE=1 SV=2 - [CCAR2_MOUSE]	Q8VDP4	C1(iodoTMT6plex); C13(iodoTMT6plex)
DVEREDIEFICK	Cct4	T-complex protein 1 subunit delta OS=Mus musculus OX=10090 GN=Cct4 PE=1 SV=3 - [TCPD_MOUSE]	P80315	C11(iodoTMT6plex)
LALEQQQLICK	Cherp	Calcium homeostasis endoplasmic reticulum protein OS=Mus musculus OX=10090 GN=Cherp PE=1 SV=1 - [CHERP_MOUSE]	Q8CGZ0	C10(iodoTMT6plex)
AVLDLVDQCPK	Cmpk2	UMP-CMP kinase 2, mitochondrial OS=Mus musculus OX=10090 GN=Cmpk2 PE=1 SV=2 - [CMPK2_MOUSE]	Q3U5Q7	C9(iodoTMT6plex)
DGIILCELINK	Cnn3	Calponin-3 OS=Mus musculus OX=10090 GN=Cnn3 PE=1 SV=1 - [CNN3_MOUSE]	Q9DAW9	C6(iodoTMT6plex)
CQQLAAYGILEK	Cops4	COP9 signalosome complex subunit 4 OS=Mus musculus OX=10090 GN=Cops4 PE=1 SV=1 - [CSN4_MOUSE]	O88544	C1(iodoTMT6plex)
YQQGDFGYCPR	Csnk2b	Casein kinase II subunit beta OS=Mus musculus OX=10090 GN=Csnk2b PE=1 SV=1 - [CSK2B_MOUSE]	P67871	C9(iodoTMT6plex)
LSVACFYGGTPYGGQIER	Ddx21	Nucleolar RNA helicase 2 OS=Mus musculus OX=10090 GN=Ddx21 PE=1 SV=3 - [DDX21_MOUSE]	Q9JIK5	C5(iodoTMT6plex)
HFILDECDK	Ddx39b	Spliceosome RNA helicase Ddx39b OS=Mus musculus OX=10090 GN=Ddx39b PE=1 SV=1 - [DX39B_MOUSE]	Q9Z1N5	C7(iodoTMT6plex)
VRPCVVYGGAEIGQQIR	Ddx3x	ATP-dependent RNA helicase DDX3X OS=Mus musculus OX=10090 GN=Ddx3x PE=1 SV=3 - [DDX3X_MOUSE]	Q62167	C4(iodoTMT6plex)
GNEFEDYCLK	Ddx6	Probable ATP-dependent RNA helicase DDX6 OS=Mus musculus OX=10090 GN=Ddx6 PE=1 SV=1 - [DDX6_MOUSE]	P54823	C8(iodoTMT6plex)
GLYDGPVCEVSVTPK	Dpysl2	Dihydropyrimidinase-related protein 2 OS=Mus musculus OX=10090 GN=Dpysl2 PE=1 SV=2 - [DPYL2_MOUSE]	O08553	C8(iodoTMT6plex)
CYLTMTQALEAR	Dync1h1	Cytoplasmic dynein 1 heavy chain 1 OS=Mus musculus OX=10090 GN=Dync1h1 PE=1 SV=2 - [DYHC1_MOUSE]	Q9JHU4	C1(iodoTMT6plex); M5(Oxidation)
ETVSEESNVLCLSK	Eef2	Elongation factor 2 OS=Mus musculus OX=10090 GN=Eef2 PE=1 SV=2 - [EF2_MOUSE]	P58252	C11(iodoTMT6plex)
GTTEEICQIYLR	Eif3c	Eukaryotic translation initiation factor 3 subunit C OS=Mus musculus OX=10090 GN=Eif3c PE=1 SV=1 - [EIF3C_MOUSE]	Q8R1B4	C7(iodoTMT6plex)
IAIWTTECENR	Eif4e	Eukaryotic translation initiation factor 4E OS=Mus musculus OX=10090 GN=Eif4e PE=1 SV=1 - [IF4E_MOUSE]	P63073	C8(iodoTMT6plex)
CAVVDVPFGGAK	Glud1	Glutamate dehydrogenase 1, mitochondrial OS=Mus musculus OX=10090 GN=Glud1 PE=1 SV=1 - [DHE3_MOUSE]	P26443	C1(iodoTMT6plex)
ITQSSLTICFPEYTGANK	Gnai2	Guanine nucleotide-binding protein G(i) subunit alpha-2 OS=Mus musculus OX=10090 GN=Gnai2 PE=1 SV=5 - [GNAI2_MOUSE]	P08752	C9(iodoTMT6plex)
ACADATLSQITNNIDPVGR	Gnb1	Guanine nucleotide-binding protein G(I)/G(S)/G(T) subunit beta-1 OS=Mus musculus OX=10090 GN=Gnb1 PE=1 SV=3 - [GBB1_MOUSE]	P62874	C2(iodoTMT6plex)
CGDLVFAK	Hdgf	Hepatoma-derived growth factor OS=Mus musculus OX=10090 GN=Hdgf PE=1 SV=2 - [HDGF_MOUSE]	P51859	C1(iodoTMT6plex)
TCNCETEDYGEK	Hnrnpu	Isoform 2 of Heterogeneous nuclear ribonucleoprotein U OS=Mus musculus OX=10090 GN=Hnrnpu - [HNRPU_MOUSE]	Q8VEK3-2	C2(iodoTMT6plex); C4(iodoTMT6plex)
AFQVWCVGNEALVGPR	Hsd17b12	Very-long-chain 3-oxoacyl-CoA reductase OS=Mus musculus OX=10090 GN=Hsd17b12 PE=1 SV=1 - [DHB12_MOUSE]	O70503	C6(iodoTMT6plex)
LECAEMDCEEGWALLK	Ifit1	Interferon-induced protein with tetratricopeptide repeats 1 OS=Mus musculus OX=10090 GN=Ifit1 PE=1 SV=2 - [IFIT1_MOUSE]	Q64282	C3(iodoTMT6plex); M6(Oxidation); C8(iodoTMT6plex)
LASAYGATELQDYCGR	Lgals3bp	Galectin-3-binding protein OS=Mus musculus OX=10090 GN=Lgals3bp PE=1 SV=1 - [LG3BP_MOUSE]	Q07797	C14(iodoTMT6plex)
CQSLTEDLEFR	Lmnb1	Lamin-B1 OS=Mus musculus OX=10090 GN=Lmnb1 PE=1 SV=3 - [LMNB1_MOUSE]	P14733	C1(iodoTMT6plex)
LVCGDHSFVK	Mnda	Isoform 2 of Interferon-activable protein 205-B OS=Mus musculus OX=10090 GN=Mnda - [IFI5B_MOUSE]	P0DOV1-2	C3(iodoTMT6plex)
HHITPLLSAVYEGHVSCVK	Mtpn	Myotrophin OS=Mus musculus OX=10090 GN=Mtpn PE=1 SV=2 - [MTPN_MOUSE]	P62774	C17(iodoTMT6plex)
KQELEEICHDLEAR	Myh9	Myosin-9 OS=Mus musculus OX=10090 GN=Myh9 PE=1 SV=4 - [MYH9_MOUSE]	Q8VDD5	C8(iodoTMT6plex)
LMTDTINEPILLCR	Nars	Asparagine--tRNA ligase, cytoplasmic OS=Mus musculus OX=10090 GN=Nars PE=1 SV=2 - [SYNC_MOUSE]	Q8BP47	M2(Oxidation); C13(iodoTMT6plex)
IISDNLTYCK	Nop58	Nucleolar protein 58 OS=Mus musculus OX=10090 GN=Nop58 PE=1 SV=1 - [NOP58_MOUSE]	Q6DFW4	C9(iodoTMT6plex)
VPFPIPEPDGCK	Npc2	NPC intracellular cholesterol transporter 2 OS=Mus musculus OX=10090 GN=Npc2 PE=1 SV=1 - [NPC2_MOUSE]	Q9Z0J0	C11(iodoTMT6plex)
CGITVSQVSNWFGNK	Pbx1	Isoform PBX1b of Pre-B-cell leukemia transcription factor 1 OS=Mus musculus OX=10090 GN=Pbx1 - [PBX1_MOUSE]	P41778-2	C1(iodoTMT6plex)
ACTGSLNMTLQR	Pdlim5	PDZ and LIM domain protein 5 OS=Mus musculus OX=10090 GN=Pdlim5 PE=1 SV=4 - [PDLI5_MOUSE]	Q8CI51	C2(iodoTMT6plex); M8(Oxidation)
EVFSSSDTDPDMAFCK	Plod3	Procollagen-lysine,2-oxoglutarate 5-dioxygenase 3 OS=Mus musculus OX=10090 GN=Plod3 PE=1 SV=1 - [PLOD3_MOUSE]	Q9R0E1	M12(Oxidation); C15(iodoTMT6plex)
DHINLPGFCGQNPLR	Pnp	Purine nucleoside phosphorylase OS=Mus musculus OX=10090 GN=Pnp PE=1 SV=2 - [PNPH_MOUSE]	P23492	C9(iodoTMT6plex)
EVGETLLYYGCR	Por	NADPH--cytochrome P450 reductase OS=Mus musculus OX=10090 GN=Por PE=1 SV=2 - [NCPR_MOUSE]	P37040	C11(iodoTMT6plex)
GISCMNTTVSESPFK	Ppa1	Inorganic pyrophosphatase OS=Mus musculus OX=10090 GN=Ppa1 PE=1 SV=1 - [IPYR_MOUSE]	Q9D819	C4(iodoTMT6plex); M5(Oxidation)
QLVTNACLIK	Prmt1	Isoform 3 of Protein arginine N-methyltransferase 1 OS=Mus musculus OX=10090 GN=Prmt1 - [ANM1_MOUSE]	Q9JIF0-3	C7(iodoTMT6plex)
YGYEIPVDMLCK	Psma6	Proteasome subunit alpha type-6 OS=Mus musculus OX=10090 GN=Psma6 PE=1 SV=1 - [PSA6_MOUSE]	Q9QUM9	M9(Oxidation); C11(iodoTMT6plex)
TTANAIYCPPK	Psmd11	26S proteasome non-ATPase regulatory subunit 11 OS=Mus musculus OX=10090 GN=Psmd11 PE=1 SV=3 - [PSD11_MOUSE]	Q8BG32	C8(iodoTMT6plex)
SSDEAVILCK	Psmd13	26S proteasome non-ATPase regulatory subunit 13 OS=Mus musculus OX=10090 GN=Psmd13 PE=1 SV=1 - [PSD13_MOUSE]	Q9WVJ2	C9(iodoTMT6plex)
GIGMNEPLVDCEGYPR	Psmd9	26S proteasome non-ATPase regulatory subunit 9 OS=Mus musculus OX=10090 GN=Psmd9 PE=1 SV=1 - [PSMD9_MOUSE]	Q9CR00	M4(Oxidation); C11(iodoTMT6plex)
VQCHEYVTEGFEK	Ptgr1	Prostaglandin reductase 1 OS=Mus musculus OX=10090 GN=Ptgr1 PE=1 SV=2 - [PTGR1_MOUSE]	Q91YR9	C3(iodoTMT6plex)
QVSTEEAQAWCK	Rab9a	Ras-related protein Rab-9A OS=Mus musculus OX=10090 GN=Rab9a PE=1 SV=1 - [RAB9A_MOUSE]	Q9R0M6	C11(iodoTMT6plex)
SPACFTLQELK	Rangap1	Ran GTPase-activating protein 1 OS=Mus musculus OX=10090 GN=Rangap1 PE=1 SV=2 - [RAGP1_MOUSE]	P46061	C4(iodoTMT6plex)
YGCLSGVR	Rcc2	Protein RCC2 OS=Mus musculus OX=10090 GN=Rcc2 PE=1 SV=1 - [RCC2_MOUSE]	Q8BK67	C3(iodoTMT6plex)
IAVHCTVR	Rpl11	60S ribosomal protein L11 OS=Mus musculus OX=10090 GN=Rpl11 PE=1 SV=4 - [RL11_MOUSE]	Q9CXW4	C5(iodoTMT6plex)
VACIGAWHPAR	Rpl3	60S ribosomal protein L3 OS=Mus musculus OX=10090 GN=Rpl3 PE=1 SV=3 - [RL3_MOUSE]	P27659	C3(iodoTMT6plex)
TGVACSVSQAQK	Rpl9	60S ribosomal protein L9 OS=Mus musculus OX=10090 GN=Rpl9 PE=2 SV=2 - [RL9_MOUSE]	P51410	C5(iodoTMT6plex)
GCTATLGNFAK	Rps2	40S ribosomal protein S2 OS=Mus musculus OX=10090 GN=Rps2 PE=1 SV=3 - [RS2_MOUSE]	P25444	C2(iodoTMT6plex)
LNISFPATGCQK	Rps6	40S ribosomal protein S6 OS=Mus musculus OX=10090 GN=Rps6 PE=1 SV=1 - [RS6_MOUSE]	P62754	C10(iodoTMT6plex)
VGSVLQEGCEK	Sdha	Succinate dehydrogenase [ubiquinone] flavoprotein subunit, mitochondrial OS=Mus musculus OX=10090 GN=Sdha PE=1 SV=1 - [SDHA_MOUSE]	Q8K2B3	C9(iodoTMT6plex)
CGGTLEER	SF3A3	Splicing factor 3A subunit 3 OS=Mus musculus OX=10090 GN=Sf3a3 PE=1 SV=2 - [SF3A3_MOUSE]	Q9D554	C1(iodoTMT6plex)
VQENCIDLVGR	SF3B1	Splicing factor 3B subunit 1 OS=Mus musculus OX=10090 GN=Sf3b1 PE=1 SV=1 - [SF3B1_MOUSE]	Q99NB9	C5(iodoTMT6plex)
GLLDVTCK	Skp1	S-phase kinase-associated protein 1 OS=Mus musculus OX=10090 GN=Skp1 PE=1 SV=3 - [SKP1_MOUSE]	Q9WTX5	C7(iodoTMT6plex)
HCNMVLENVK	Snrpd2	Small nuclear ribonucleoprotein Sm D2 OS=Mus musculus OX=10090 GN=Snrpd2 PE=1 SV=1 - [SMD2_MOUSE]	P62317	C2(iodoTMT6plex); M4(Oxidation)
CLHMFLQEEAIDR	Snx12	Sorting nexin-12 OS=Mus musculus OX=10090 GN=Snx12 PE=1 SV=1 - [SNX12_MOUSE]	O70493	C1(iodoTMT6plex); M4(Oxidation)
CNSLEEIK	Sptan1	Isoform 2 of Spectrin alpha chain, non-erythrocytic 1 OS=Mus musculus OX=10090 GN=Sptan1 - [SPTN1_MOUSE]	P16546-2	C1(iodoTMT6plex)
LGLIQEDVASSCIPR	Srrm2	Isoform 3 of Serine/arginine repetitive matrix protein 2 OS=Mus musculus OX=10090 GN=Srrm2 - [SRRM2_MOUSE]	Q8BTI8-3	C12(iodoTMT6plex)
LIGPNCPGVINPGECK	Suclg1	Succinate--CoA ligase [ADP/GDP-forming] subunit alpha, mitochondrial OS=Mus musculus OX=10090 GN=Suclg1 PE=1 SV=4 - [SUCA_MOUSE]	Q9WUM5	C6(iodoTMT6plex); C15(iodoTMT6plex)
CDFAYETMGTIEVQR	Tomm70	Mitochondrial import receptor subunit TOM70 OS=Mus musculus OX=10090 GN=Tomm70 PE=1 SV=2 - [TOM70_MOUSE]	Q9CZW5	C1(iodoTMT6plex); M8(Oxidation)
CVFEMPNENDK	Vapa	Vesicle-associated membrane protein-associated protein A OS=Mus musculus OX=10090 GN=Vapa PE=1 SV=2 - [VAPA_MOUSE]	Q9WV55	C1(iodoTMT6plex); M5(Oxidation)
DCPVPLPGDGDLLVR	Zadh2	Prostaglandin reductase-3 OS=Mus musculus OX=10090 GN=Zadh2 PE=1 SV=1 - [PTGR3_MOUSE]	Q8BGC4	C2(iodoTMT6plex)
IPGGIIEDSCVLR	Cct3	T-complex protein 1 subunit gamma OS=Mus musculus OX=10090 GN=Cct3 PE=1 SV=1 - [TCPG_MOUSE]	P80318	C10(iodoTMT6plex)
DQGSCGSCWAFGAVEAISDR	Ctsb	Cathepsin B OS=Mus musculus OX=10090 GN=Ctsb PE=1 SV=2 - [CATB_MOUSE]	P10605	C5(iodoTMT6plex); C8(iodoTMT6plex)
TCIHTNGR	Ctsb	Cathepsin B OS=Mus musculus OX=10090 GN=Ctsb PE=1 SV=2 - [CATB_MOUSE]	P10605	C2(iodoTMT6plex)
AEHCVNTPGSFQCYK	Fbln2	Isoform 2 of Fibulin-2 OS=Mus musculus OX=10090 GN=Fbln2 - [FBLN2_MOUSE]	P37889-2	C4(iodoTMT6plex); C13(iodoTMT6plex)
CSCAAGFLLAADGK	Fbln2	Isoform 2 of Fibulin-2 OS=Mus musculus OX=10090 GN=Fbln2 - [FBLN2_MOUSE]	P37889-2	C1(iodoTMT6plex); C3(iodoTMT6plex)
CGEGQLCYNLPGSYR	Fbln2	Isoform 2 of Fibulin-2 OS=Mus musculus OX=10090 GN=Fbln2 - [FBLN2_MOUSE]	P37889-2	C1(iodoTMT6plex); C7(iodoTMT6plex)
CSGPGLSPGMVR	Flna	Filamin-A OS=Mus musculus OX=10090 GN=Flna PE=1 SV=5 - [FLNA_MOUSE]	Q8BTM8	C1(iodoTMT6plex); M10(Oxidation)
SNFTVDCSK	Flna	Filamin-A OS=Mus musculus OX=10090 GN=Flna PE=1 SV=5 - [FLNA_MOUSE]	Q8BTM8	C7(iodoTMT6plex)
ACIPQSFTVDSSK	Flnb	Filamin-B OS=Mus musculus OX=10090 GN=Flnb PE=1 SV=3 - [FLNB_MOUSE]	Q80X90	C2(iodoTMT6plex)
IAGPGLSSCVR	Flnb	Filamin-B OS=Mus musculus OX=10090 GN=Flnb PE=1 SV=3 - [FLNB_MOUSE]	Q80X90	C9(iodoTMT6plex)
CFTDEPQLQSR	Gvin1	Interferon-induced very large GTPase 1 OS=Mus musculus OX=10090 GN=Gvin1 PE=1 SV=1 - [GVIN1_MOUSE]	Q80SU7	C1(iodoTMT6plex)
CWEVSDPEEQLTK	Gvin1	Interferon-induced very large GTPase 1 OS=Mus musculus OX=10090 GN=Gvin1 PE=1 SV=1 - [GVIN1_MOUSE]	Q80SU7	C1(iodoTMT6plex)
QITQVYGFYDECLR	Ppp2ca	Serine/threonine-protein phosphatase 2A catalytic subunit alpha isoform OS=Mus musculus OX=10090 GN=Ppp2ca PE=1 SV=1 - [PP2AA_MOUSE	P63330	C12(iodoTMT6plex)
AHQLVMEGYNWCHDR	Ppp2ca	Serine/threonine-protein phosphatase 2A catalytic subunit alpha isoform OS=Mus musculus OX=10090 GN=Ppp2ca PE=1 SV=1 - [PP2AA_MOUSE	P63330	M6(Oxidation); C12(iodoTMT6plex)
SEGTYCCGPVSVR	Tgm2	Protein-glutamine gamma-glutamyltransferase 2 OS=Mus musculus OX=10090 GN=Tgm2 PE=1 SV=4 - [TGM2_MOUSE]	P21981	C6(iodoTMT6plex); C7(iodoTMT6plex)
VVSAMVNCNDDQGVLLGR	Tgm2	Protein-glutamine gamma-glutamyltransferase 2 OS=Mus musculus OX=10090 GN=Tgm2 PE=1 SV=4 - [TGM2_MOUSE]	P21981	M5(Oxidation); C8(iodoTMT6plex)
VVAPTISSPVCQEQLVEAGR	Tln1	Talin-1 OS=Mus musculus OX=10090 GN=Tln1 PE=1 SV=2 - [TLN1_MOUSE]	P26039	C11(iodoTMT6plex)
AGALQCSPSDVYTK	Tln1	Talin-1 OS=Mus musculus OX=10090 GN=Tln1 PE=1 SV=2 - [TLN1_MOUSE]	P26039	C6(iodoTMT6plex)
CVSCLPGQR	Tln1	Talin-1 OS=Mus musculus OX=10090 GN=Tln1 PE=1 SV=2 - [TLN1_MOUSE]	P26039	C1(iodoTMT6plex); C4(iodoTMT6plex)
QVAASTAQLLVACK	Tln1	Talin-1 OS=Mus musculus OX=10090 GN=Tln1 PE=1 SV=2 - [TLN1_MOUSE]	P26039	C13(iodoTMT6plex)

Total 93 oxidized Cys in 82 proteins (Cct3 contains both oxidized and reduced Cys)

SUPPLEMENTAL TABLE 1B: Proteins with cysteines whose redox state became more reduced with aging and restored by Slc7a11 overexpression

Sequence	Protein	Description	Protein Group Accessions	Modifications
LCYVALDFEQEMATAASSSSLEK	Actb	Actin, cytoplasmic 1 OS=Mus musculus OX=10090 GN=Actb PE=1 SV=1 - [ACTB_MOUSE]	P60710	C2(iodoTMT6plex); M12(Oxidation)
GLGTDEDSLIEIICSR	Anxa2	Annexin A2 OS=Mus musculus OX=10090 GN=Anxa2 PE=1 SV=2 - [ANXA2_MOUSE]	P07356	C14(iodoTMT6plex)
ECTIEATA	Cnbp	Isoform 2 of Cellular nucleic acid-binding protein OS=Mus musculus OX=10090 GN=Cnbp - [CNBP_MOUSE]	P53996-2	C2(iodoTMT6plex)
VADPVVTFCETVVETSSLK	Eftud2	116 kDa U5 small nuclear ribonucleoprotein component OS=Mus musculus OX=10090 GN=Eftud2 PE=1 SV=1 - [U5S1_MOUSE]	O08810	C9(iodoTMT6plex)
FMCAQLPNPVLDSISIIDTPGILSGEK	Ehd1	EH domain-containing protein 1 OS=Mus musculus OX=10090 GN=Ehd1 PE=1 SV=1 - [EHD1_MOUSE]	Q9WVK4	M2(Oxidation); C3(iodoTMT6plex)
IVLTNPVCTEVGEK	Eif2s3x	Eukaryotic translation initiation factor 2 subunit 3, X-linked OS=Mus musculus OX=10090 GN=Eif2s3x PE=1 SV=2 - [IF2G_MOUSE]	Q9Z0N1	C8(iodoTMT6plex)
THGLSDEHVFEVICPSIPGYGFSEASSK	Ephx1	Epoxide hydrolase 1 OS=Mus musculus OX=10090 GN=Ephx1 PE=1 SV=2 - [HYEP_MOUSE]	Q9D379	C14(iodoTMT6plex)
DGSCGVSYVVQEPGDYEVSIK	Flnc	Isoform 2 of Filamin-C OS=Mus musculus OX=10090 GN=Flnc - [FLNC_MOUSE]	Q8VHX6-2	C4(iodoTMT6plex)
ACAETIDPASLWEY	Fscn1	Fascin OS=Mus musculus OX=10090 GN=Fscn1 PE=1 SV=4 - [FSCN1_MOUSE]	Q61553	C2(iodoTMT6plex)
ASADLMSYCEEHAR	Gng12	Guanine nucleotide-binding protein G(I)/G(S)/G(O) subunit gamma-12 OS=Mus musculus OX=10090 GN=Gng12 PE=1 SV=3 - [GBG12_MOUSE]	Q9DAS9	M6(Oxidation); C9(iodoTMT6plex)
TNQPLSTC	Hars	Histidine--tRNA ligase, cytoplasmic OS=Mus musculus OX=10090 GN=Hars PE=1 SV=2 - [SYHC_MOUSE]	Q61035	C8(iodoTMT6plex)
CELSSSVQTDINLPYLTMDASGPK	Hspa9	Stress-70 protein, mitochondrial OS=Mus musculus OX=10090 GN=Hspa9 PE=1 SV=3 - [GRP75_MOUSE]	P38647	C1(iodoTMT6plex); M18(Oxidation)
CATITPDEK	Idh1	Isocitrate dehydrogenase [NADP] cytoplasmic OS=Mus musculus OX=10090 GN=Idh1 PE=1 SV=2 - [IDHC_MOUSE]	O88844	C1(iodoTMT6plex)
FFQVACDVPELQDK	Iqgap1	Ras GTPase-activating-like protein IQGAP1 OS=Mus musculus OX=10090 GN=Iqgap1 PE=1 SV=2 - [IQGA1_MOUSE]	Q9JKF1	C6(iodoTMT6plex)
YNLGAPVAGTCYQAEWDDYVPK	Pebp1	Phosphatidylethanolamine-binding protein 1 OS=Mus musculus OX=10090 GN=Pebp1 PE=1 SV=3 - [PEBP1_MOUSE]	P70296	C11(iodoTMT6plex)
VCTQLQVLTEAFR	Pef1	Peflin OS=Mus musculus OX=10090 GN=Pef1 PE=1 SV=1 - [PEF1_MOUSE]	Q8BFY6	C2(iodoTMT6plex)
ICIVTK	Psmb1	Proteasome subunit beta type-1 OS=Mus musculus OX=10090 GN=Psmb1 PE=1 SV=1 - [PSB1_MOUSE]	O09061	C2(iodoTMT6plex)
QLQNQLAECK	Rai14	Ankycorbin OS=Mus musculus OX=10090 GN=Rai14 PE=1 SV=1 - [RAI14_MOUSE]	Q9EP71	C9(iodoTMT6plex)
CAEAGANMIVSGSAIMR	Rpe	Ribulose-phosphate 3-epimerase OS=Mus musculus OX=10090 GN=Rpe PE=1 SV=1 - [RPE_MOUSE]	Q8VEE0	C1(iodoTMT6plex); M8(Oxidation); M16(Oxidation)
ALAGCDFLTISPK	Taldo1	Transaldolase OS=Mus musculus OX=10090 GN=Taldo1 PE=1 SV=2 - [TALDO_MOUSE]	Q93092	C5(iodoTMT6plex)
SQLCSQSLEITR	Uso1	Isoform 4 of General vesicular transport factor p115 OS=Mus musculus OX=10090 GN=Uso1 - [USO1_MOUSE]	Q9Z1Z0-4	C4(iodoTMT6plex)
MEDSGVYVCEGVNLIGR	Vcam1	Vascular cell adhesion protein 1 OS=Mus musculus OX=10090 GN=Vcam1 PE=1 SV=1 - [VCAM1_MOUSE]	P29533	M1(Oxidation); C9(iodoTMT6plex)
IGDEYFTFITDCK	Cct3	T-complex protein 1 subunit gamma OS=Mus musculus OX=10090 GN=Cct3 PE=1 SV=1 - [TCPG_MOUSE]	P80318	C12(iodoTMT6plex)

Total 23 reduced Cys in 23 proteins (Cct3 contains both oxidized and reduced Cys)

Total 104 proteins with age-dependent Slc7a11-responsive Cys

Supplemental Table 2 provides IPA analysis of proteins whose redox state changes with Slc7a11 overexpression in old fibroblasts regardless of their redox states in young fibroblasts. Cysteine redox states were compared between old fibroblasts and old Slc7a11-overexpressing fibroblasts. This table is described in the Results section 3.2.

SUPPLEMENTAL TABLE 2: IPA analysis of proteins whose redox states were changed by Slc7a11 overexpression in old fibroblasts. Fisher's exact test *p*-values for all the items shown were less than 0.05.

Name	p-value	Overlap
**Regulation of eIF4 and p70S6K Signaling**	2.09E-09	6.4 % 10/157
**EIF2 Signaling**	6.26E-08	4.5 % 10/224
**mTOR Signaling**	4.53E-06	3.8 % 8/210
**Antiproliferative Role of Somatostatin Receptor 2**	2.41E-05	6.5 % 5/77
**Relaxin Signaling**	5.68E-05	4.0 % 6/150

Supplemental Table 3 provides IPA analysis of age-dependent proteins whose redox states were not rescued by Slc7a11 overexpression in old fibroblasts. Those age-dependent Slc7a11-nonresponsive protein cysteines indicate there are Slc7a11-independent regulation of protein redox signaling. This table is described in the Results section 3.3.

SUPPLEMENTAL TABLE 3: IPA analysis of age-dependent proteins whose redox states were not rescued by Slc7a11 overexpression in old fibroblasts. Fisher's exact test *p*-values for all the items shown were less than 0.05.

Name	p-value	Overlap	
**Hepatic Fibrosis / Hepatic Stellate Cell Activation**	3.27E-05	2.7 %	5/186
**Protein Ubiquitination Pathway**	2.00E-04	1.8 %	5/273
**Epithelial Adherens Junction Signaling**	2.32E-04	2.6 %	4/152
**Actin Cytoskeleton Signaling**	9.04E-04	1.8 %	4/218
**EIF2 Signaling**	1.00E-03	1.8 %	4/224

Supplemental Table 4 provides GO-based biological process analysis of proteins whose redox states were not rescued by Slc7a11 overexpression in old fibroblasts. Those age-dependent Slc7a11-nonresponsive protein cysteines indicate there are Slc7a11-independent regulation of protein redox signaling. This table is described in the Results section 3.3.

SUPPLEMENTAL TABLE 4: GO-based biological process analysis of proteins whose redox states were not rescued by Slc7a11 overexpression in old fibroblasts. Fisher's exact test *p*-values for all the items shown were less than 0.05.

Term	Count	p-value	-log10 (p-value)
protein folding	6	<0.001	4.845
wound healing	4	0.002	2.811
actomyosin structure organization	3	0.002	2.709
mitotic cytokinesis	3	0.002	2.618
translation	6	0.003	2.554
skin development	3	0.009	2.057
IRES-dependent viral translational initiation	2	0.017	1.772
platelet degranulation	2	0.019	1.714
heart development	4	0.025	1.594
cytoskeleton organization	3	0.026	1.577
single organismal cell-cell adhesion	3	0.028	1.547
cerebellar Purkinje cell layer development	2	0.038	1.417
actin filament-based movement	2	0.041	1.392
actin filament capping	2	0.043	1.367
